# Polymerization-Induced Self-Assembly for Efficient Fabrication of Biomedical Nanoplatforms

**DOI:** 10.34133/research.0113

**Published:** 2023-04-11

**Authors:** Xiaopeng Zhao, Changrui Sun, Fei Xiong, Ting Wang, Sheng Li, Fengwei Huo, Xikuang Yao

**Affiliations:** School of Flexible Electronics (Future Technologies) and Institute of Advanced Materials (IAM), Nanjing Tech University (NanjingTech), Nanjing 211816, P. R. China.

## Abstract

Amphiphilic copolymers can self-assemble into nano-objects in aqueous solution. However, the self-assembly process is usually performed in a diluted solution (<1 wt%), which greatly limits scale-up production and further biomedical applications. With recent development of controlled polymerization techniques, polymerization-induced self-assembly (PISA) has emerged as an efficient approach for facile fabrication of nano-sized structures with a high concentration as high as 50 wt%. In this review, after the introduction, various polymerization method-mediated PISAs that include nitroxide-mediated polymerization-mediated PISA (NMP-PISA), reversible addition-fragmentation chain transfer polymerization-mediated PISA (RAFT-PISA), atom transfer radical polymerization-mediated PISA (ATRP-PISA), and ring-opening polymerization-mediated PISA (ROP-PISA) are discussed carefully. Afterward, recent biomedical applications of PISA are illustrated from the following aspects, i.e., bioimaging, disease treatment, biocatalysis, and antimicrobial. In the end, current achievements and future perspectives of PISA are given. It is envisioned that PISA strategy can bring great chance for future design and construction of functional nano-vehicles.

## Introduction

In recent years, nanoparticles (NPs) that can be precisely designed and manipulated have been applied in a range of fields including catalysis, energy storage and conversion, and drug delivery [1–7]. Polymeric NPs are appealing materials for biomedical applications owing to their excellent biocompatibility and tailorability. Particularly, amphiphilic copolymers can simultaneously self-assemble into various nanostructures in aqueous solution [8]. Notably, Zhang and Eisenberg first reported the self-assembly behavior of amphiphilic block copolymers (BCP) in solution in 1995 [9]. Since then, various NPs including spheres, worms, and vesicles have been discovered [10–12]. However, the process is usually performed in dilute solution (<1 wt%). The efficiency and reproducibility of the preparation of polymeric NPs are unpleasant. These above adverse defects greatly restrict the large-scale preparation of functional NPs and their applications. Therefore, it is quite demanding to find an alternative approach to prepare NPs efficiently.

Notably, polymerization-induced self-assembly (PISA) that combines polymerization and self-assembly in one pot has shown many distinct advantages, such as labor-saving, repeatability, and high solid concentration as high as 50 wt% [13–17]. By utilization of suitable solvent, soluble macroinitiator can be chain-extended by polymerization with the second monomers. As the polymerization proceeds, the second insoluble block gradually drives the self-assembly of polymer chains. Armes and coworkers summarized the PISA process under the different conditions [18,19]. The morphology of obtained NPs can be well regulated during the PISA process [20–22]. Notably, the morphology of self-assembled NPs can be finely tuned by adjusting the packing parameter (*P*). *P* can be given by the equation *P* = *v*/*al*, where *a* is the interfacial area of the hydrophilic block and *v* and *l* are the volume and length of the hydrophobic block, respectively. When *P* ≤ 1/3, amphiphilic BCPs usually self-assemble into spherical micelles. Worms can be obtained when 1/3 < *P* ≤ 1/2, while vesicles are formed when 1/2 < *P* ≤ 1. Interestingly, when *P* > 1, highly-ordered inverse structures (e.g., spongosome and cubosome) can be fabricated. Pan and Du reported that the PISA process has been succeeded by utilization of controlled polymerization techniques including nitroxide-mediated radical polymerization (NMP), atom transfer radical polymerization (ATRP), reversible addition-fragmentation chain transfer (RAFT) polymerization, and ring-opening polymerization (ROP) [23,24].

Due to fine control over the morphology and size of the prepared NPs, PISA has become a robust technique to fabricate NPs for various applications. As a new trend, growing functional groups have been gradually introduced into the PISA system to construct multifunctional nanoplatforms [25–28]. Notably, by incorporation of reactive groups, such as thiol group [29] and epoxy group [30], into hydrophilic chain or hydrophobic core, fabricated NPs can further conjugate with functional molecules. What is more, theranostic molecules, such as imaging agents, drugs, and proteins, can also be finely encapsulated during the self-assembly process, resulting in enhanced diagnostic and therapeutic effects. In addition, enzymes with catalytic effect can also be combined with polymeric nanoreactors to achieve biocatalysis and antimicrobial action. Recently, these polymeric NPs manufactured by PISA have been emerged in biomedical field.

Recently, PISA has been reviewed by Armes, O’Reilly, Pan, Yuan, Couturaud, and Du, as well as other groups from various perspectives [19,23,24,31–34]. Among these existing reviews, Pan and Du recently summarized the development of polymerization techniques in PISA [23,24]. Yuan and Armes focused on PISA by nonthermal initiation, which was beneficial to the loading of biomacromolecules [14,33]. Couturaud and Pan also reviewed the application of PISA for drug delivery [32,34]. However, none of them focused on the fabrication of nanoplatforms by PISA for biomedical applications. As an efficient tool to prepare functional NPs, fabricating biomedical nanoplatforms by PISA is worthy of much attention.

**Fig. 1.**
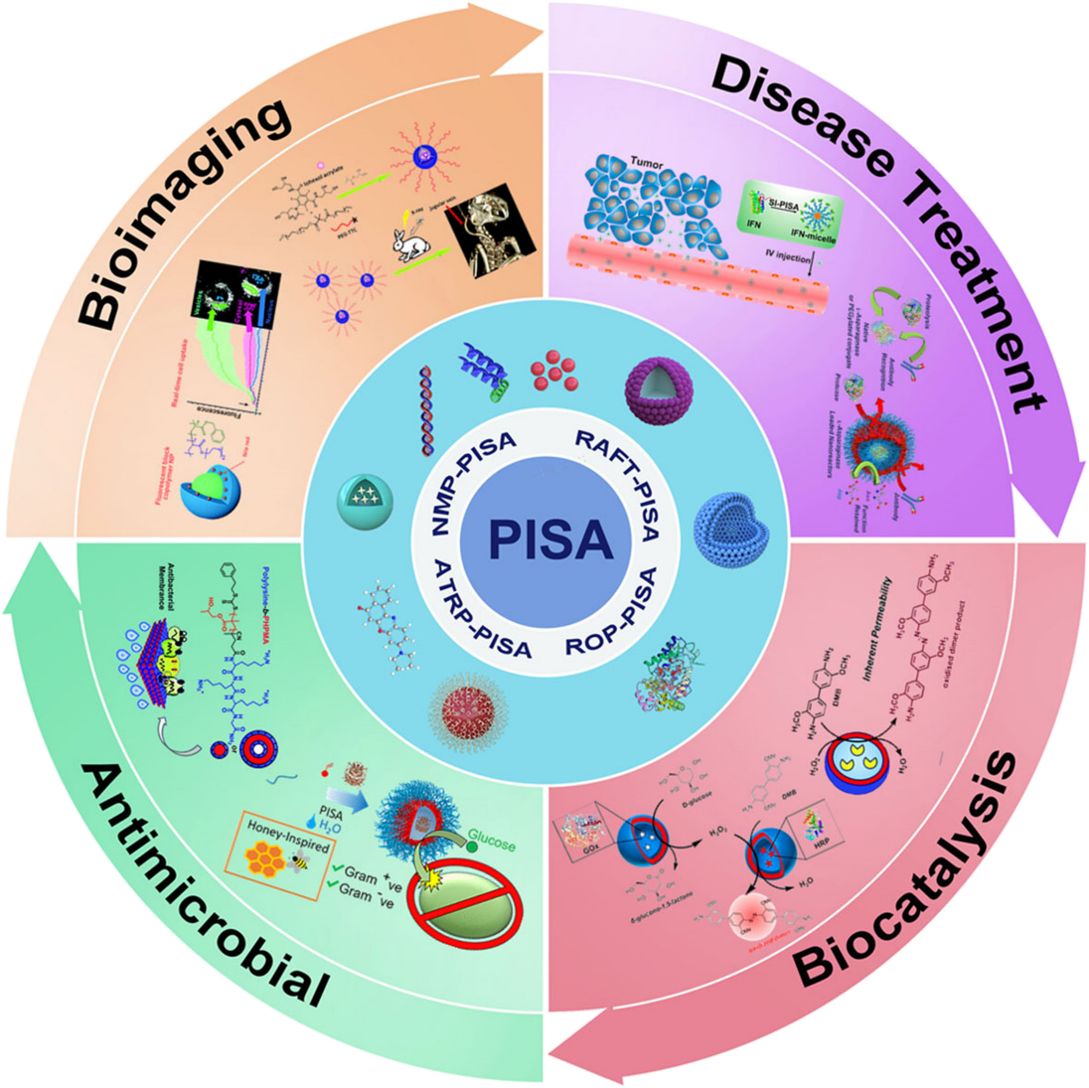
Fabrication of nanoplatforms by polymerization-induced self-assembly and their biomedical applications. Reproduced with permission from [136]. Copyright 2018, Royal Society of Chemistry. Reproduced with permission from [143]. Copyright 2022, Royal Society of Chemistry. Reproduced with permission from [159]. Copyright 2018, American Chemical Society. Reproduced with permission from [164]. Copyright 2017, American Chemical Society. Reproduced with permission from [169]. Copyright 2020, American Chemical Society. Reproduced with permission from [170]. Copyright 2019, Royal Society of Chemistry.

In this review, after the introduction, the principles and features of various PISA systems are demonstrated with depth (Fig. 1). Afterward, updated biomedical nanoplatforms built by PISA strategy are reviewed from 4 aspects, i.e., bioimaging, disease treatment, biocatalysis, and antimicrobial. In the end, some conclusions and perspectives for further development of PISA are elaborated carefully. It is believed that more and more fascinating polymeric nanoplatforms for other applications can be constructed by PISA in the future.

**Table 1.** Comparison of different PISA formulations.

PISA formulation	Reaction temperature	Solvent	Advantages	Disadvantages	Reference
NMP-PISA	85–120 °C	Water, 1,4-dioxane, EtOH	Without sulfur-containing end groups	High reaction temperature and uncommon agents	[35–40]
Thermal-initiated RAFT-PISA	60–90 °C	Polar and nonpolar solvents	Well-established and widespread applicability	The biotoxicity of sulfur-containing end groups, high reaction temperature and nondegradable backbone	[55–66]
Photo-initiated RAFT-PISA	RT	Water, MeOH	Mild reaction condition	Limited light penetration depth	[71–85]
Enzyme-initiated RAFT-PISA	RT	Water	Mild reaction condition	Limited solvent for reaction	[89,94]
Sono-initiated RAFT-PISA	RT	Water	Without additional initiator	Limited solvent for reaction	[97,98]
ATRP-PISA	RT to 85 °C	Water/MeOH, scCO_2_	Without sulfur-containing end groups	The potential toxicity of copper catalyst	[104–112]
ROMP-PISA	RT to 40 °C	Water, toluene	Excellent tolerance to reactive groups	Limited reactive monomers and removal of Ru-based catalysts	[114–121]
ROPISA with NCA monomers	4–10 °C	THF, water	Excellent biocompatibility and biodegradation	Monomer storage and limited morphology	[126–129]
rROPISA	90 °C	DMF, heptane	Attractive degradability	Biotoxicity of sulfur-containing species and high reaction temperature	[130,131]

## Preparation and Characterization of Nanoplatforms by PISA

### NMP-PISA

**Fig. 2.**
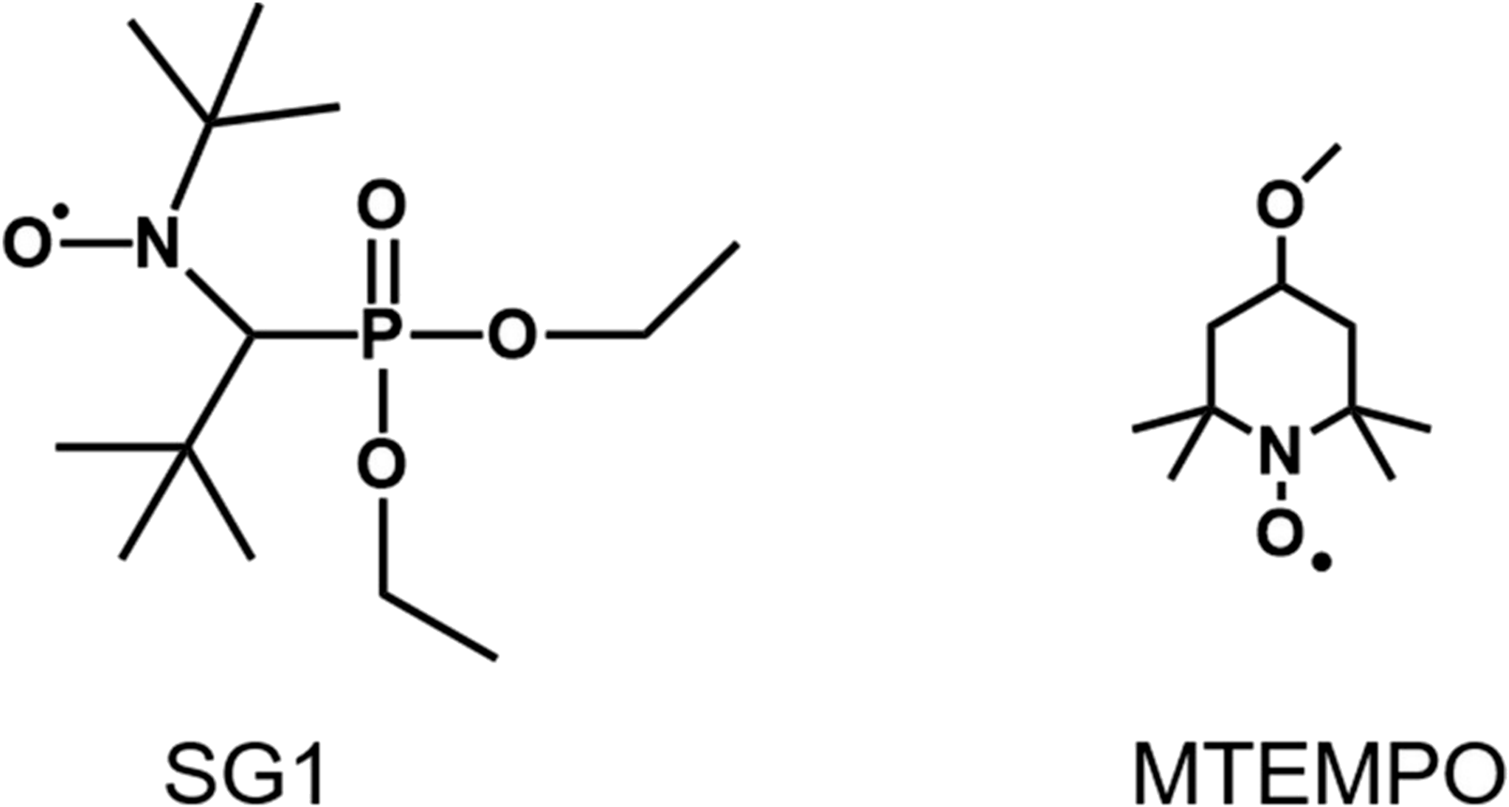
Chemical structure of initiators applied in NMP-PISA.

NMP is one of the earliest discovered reversible-deactivation radical polymerization (RDRP) techniques, and the reaction process can achieve controlled/active polymerization by the utilization of 2,2,6,6-tetramethylpiperidine (TEMPO) or its derivatives (Fig. 2). The first work about NMP-PISA was reported in 2005 by Charleux’s group [35]. Briefly, styrene (St) and *n*-butyl acrylate (BA) monomers were grafted from the end of *N*-*tert*-butyl-*N*-(1-diethyl phosphono-2,2-dimethyl propyl) nitroxide (SG1)-terminated poly(sodium acrylate) in water at 120 °C under 3 bar pressure of nitrogen. With the growth of hydrophobic chain, obtained amphiphilic BCPs self-assembled into spherical NPs in situ. The hydrodynamic diameter of polystyrene (PS) and poly(*n*-butyl acrylate) (PBA) particles was 65 and 90 nm, respectively. Notably, when pH was varied from 7 to 4, the diameters of PS NPs decreased to 55 nm and the diameters of PBA NPs decreased to 76 nm. Notably, the variation of the diameters was due to the collapse of hydrophilic corona after protonation. This pioneering work laid the foundation for future research.

**Table 2.** Summary of the stabilizer block, core-forming block, PISA formulation, corresponding morphology, and the biomedical application.

Stabilizer block	Core-forming block	PISA formulation	Morphology	Application	Reference
PEG	P(BMA-*co*-BzMA)	RAFT-PISA	S/V	Bioimaging	[129]
POEGA	PBZA	RAFT-PISA	S	Bioimaging	[128]
PEG	PHPMA	RAFT-PISA	S	Bioimaging	[121]
PEG	PStCH	RAFT-PISA	S	Bioimaging	[127]
PEG	PTFEAM-PHEAM	RAFT-PISA	S	Bioimaging	[123]
POEGMA	P(ST-*co*-VBA)	RAFT-PISA	S/W/V	Treatment	[133]
PDMAEMA	PMAEBA	RAFT-PISA	S/W/V	Treatment	[134]
PEG-b-P(MEO_2_MA-co-CPTM)	BzMA	RAFT-PISA	S	Treatment	[136]
P(OEG)	PCAND-PDEND	ROMP-PISA	S	Treatment	[140]
POEGMA	PHPMA	ATRP-PISA	S	Treatment	[143]
PEG	PHPMA	RAFT-PISA	V	Treatment	[144]
PGMA	PHPMA-PDPA	RAFT-PISA	V	Treatment	[145]
PEG	P(HPMA-*co*-GlyMA)	RAFT-PISA	V	Biocatalysis	[149]
PEG	PHPMA	RAFT-PISA	V	Biocatalysis	[148]
PEG	PHPMA	RAFT-PISA	V	Antimicrobial	[151]
polylysine	PHPMA	RAFT-PISA	S/W/V	Antimicrobial	[152]

S, spherical micelle; W, wormlike micelle; V, vesicle

Later in 2009, the same group first reported pH-sensitive vesicles, which were fabricated with high concentrations by NMP-PISA under emulsion condition [36]. Poly(sodium acrylate) macroalkoxyamine (PNaA-SG1) was used to polymerize with 4-vinylpyridine (4VP) monomers at pH 11, 120 °C under 3 bar pressure. Most monomers (>90%) were consumed within 4 h, as the polymerization proceeded, and spheres, wormlike micelles, and spherical vesicles were observed. When the pH decreased from 11 to 2, the milky solution became clear, ascribed to the protonation of poly(4-vinylpyridine) (P4VP) block. Therefore, these pH-sensitive vesicles could efficiently release encapsulated drugs once upon acid environment. However, the disadvantages of NMP also existed in the above PISA process including high reaction temperature, which was not beneficial to the encapsulation of biomolecules.

To simplify the NMP-PISA process, some pioneering works have been reported. For this purpose, water-soluble brush-type macroalkoxyamine initiator poly (poly (ethylene oxide) methyl ether methacrylate*-co-*styrene) was used to initiate emulsion polymerization of *n*-butyl methacrylate and St units at 85 °C [37]. The effect of the macroinitiator concentration, pH, and the salt concentration on the stability and morphology of self-assembled nano-objects were studied. If salt concentration was at 0 or 1 mM, spherical NPs tended to be obtained. When salt concentration was increased to 10 mM or even 100 mM, elongated micelles and vesicles appeared. It was explained that the polymer became more hydrophilic with increased ionic strength, which could induce the transformation of the morphology. Yoshida fabricated giant vesicles by NMP-PISA under room temperature (RT) and ultraviolet (UV) irradiation instead of heating [38,39].

In addition to the study of polymeric assemblies, NMP-PISA can also be used for the fabrication of polymer–inorganic hybrid systems [40]. For this purpose, macroalkoxyamine initiator was adsorbed on the surface of silica NPs to copolymerize with *n*-butyl methacrylate (BMA) and St units by NMP. As the hydrophobic block grew, amphiphilic BCPs self-assembled into various morphologies. Impressively, both the pH value and the size of silica NPs had important impact on the morphology of nanocomposites. Notably, by taking advantage of the cryo-transmission electron microscope (TEM) technique, the intermediate morphologies during the PISA process were captured, which was beneficial to understand the mechanism of the self-assembly behaviors.

Above all, although NMP-PISA has been explored for many years, the literatures that have been reported remain sparse as a result of stringent experimental conditions and uncommon agents. Meanwhile, the reported monomers for NMP-PISA are quite limited and possess poor biocompatibility and biodegradability.

### RAFT-PISA

Among the controllable radical polymerization techniques, RAFT polymerization has been considered as a robust method to prepare well-defined polymers with desired structure and molecular weight (MW). Generally, the polymerization process usually requires the usage of chain transfer agents (CTAs), such as dithioester derivatives [RC(=S)-Z]. In recent years, much effort has been used to develop RAFT-PISA [41,42]. In general, solvophilic macro-CTAs should be first synthesized as stabilizer block and chain-extended with the solvophobic block. With the increase of the solvophobic chain, amphiphilic BCPs self-assemble into NPs in situ.

In general, RAFT-PISA can be performed in various kinds of solvents, including polar solvents [43–48] and nonpolar solvents [49–54]. Also, there are abundant monomers that can be suitable for RAFT-PISA, such as vinyl-based monomers and methacrylates. In the following, RAFT-PISA will be introduced carefully according to initiation method.

#### Thermal-initiated RAFT-PISA

Among all these RAFT-PISA systems, thermal initiation is the most well-established initiation method. Traditional initiators [e.g., 2,2'-azobisisobutyronitrile ( AIBN) or 4,4'-azobis(4-cyanovaleric acid) (ACVA)] can produce free radicals at high temperature (60 to 90 °C), which will initiate the polymerization process. Also, various kinds of amphiphilic copolymer-based NPs have been successfully fabricated by thermal-initiated RAFT-PISA in dispersion or emulsion condition [52,55–58]. The fabrication of these NPs can be predicted and guided by phase diagram [59,60].

Nowadays, ,many works were devoted to control the morphology of obtained NPs. In addition to the significant effects of solid content and chain length on the morphology of NPs, other factors such as monomer solubility, temperature, pH, and complementary hydrogen bonding interaction were also studied [61–65]. Very recently, the effect of host–guest complexation interaction on the morphology has attracted much attention. For example, Yuan and coworkers first achieved aqueous dispersion polymerization with water-immiscible monomers by using host–guest chemistry [46]. The ratio of cyclodextrin/styrene (CD/St) was regulated to obtain various morphologies. When the CD/St molar ratio was set as 1:1, the dumbbell structure was observed with the increased degree of polymerization (DP) of PS block. By using host–guest chemistry, the library of monomers for RAFT-PISA is greatly expanded. More recently, the same group successfully fabricated uniform polyethylene glycol (PEG)*-b-*PSt nanoflowers with low dispersity by increasing the polymerization rate [45]. This strategy could also be applied to other PISA formulations to fabricate monodisperse and hierarchical NPs. In addition, Armes and coworkers fabricated PEG-decorated vesicles by utilization of host–guest interaction between azobenzene-capped PEG and CD-modified vesicles to regulate the kinetics of morphological transition [66]. It was found that these supramolecular vesicles had a faster response to temperature than that of pure CD-modified vesicles, which may have great potential in design and fabrication of light-controlled drug delivery systems.

Although the thermal initiation is commonly used, the relatively high temperature involved in the reaction process limits the scope of RAFT-PISA. High temperature hampers the introduction of some biological materials (e.g., enzyme), which are labile to heat. Therefore, exploring more moderate reaction conditions and simpler steps is a trend of PISA development. Nonthermally initiated RAFT-PISA formulations (e.g., photo, enzyme, and ultrasound wave initiation) seem to have great potential in bioapplications.

#### Photo-initiated RAFT-PISA

Photo-initiated RAFT polymerization has attracted much attention owing to its robust control of polymerization in time and space [67–70]. Compared with thermal initiation, photo-initiation can allow the polymerization process to take place at low temperature. According to the wavelength of light, photoinitiation mainly includes UV and visible light initiation. Photo-initiated RAFT polymerization is mild because it is usually conducted at RT.

Notably, Chen and coworkers creatively introduced photo-initiated RAFT polymerization into the PISA system [71]. The macro-CTA P4VP was chain-extended with St units in methanol upon UV light, resulting in polymeric micelles at ambient temperature. However, the polymerization kinetics showed that the monomer conversion was relatively low in this photo-initiated polymerization process when compared with thermal-initiated dispersion polymerization, which was attributed to low activity of St at RT. In addition, when the MW did not increase upon UV light, the reaction could achieve secondary polymerization by heating. This interesting phenomenon may be explained that some encapsulated photoinitiator like AIBN cannot be irradiated by UV light due to the limited penetration depth. In general, the photo-initiated RAFT-PISA expands the scope of PISA formulations and shows promise as a powerful strategy to scale up preparation of polymeric NPs under mild conditions.

**Fig. 3.**
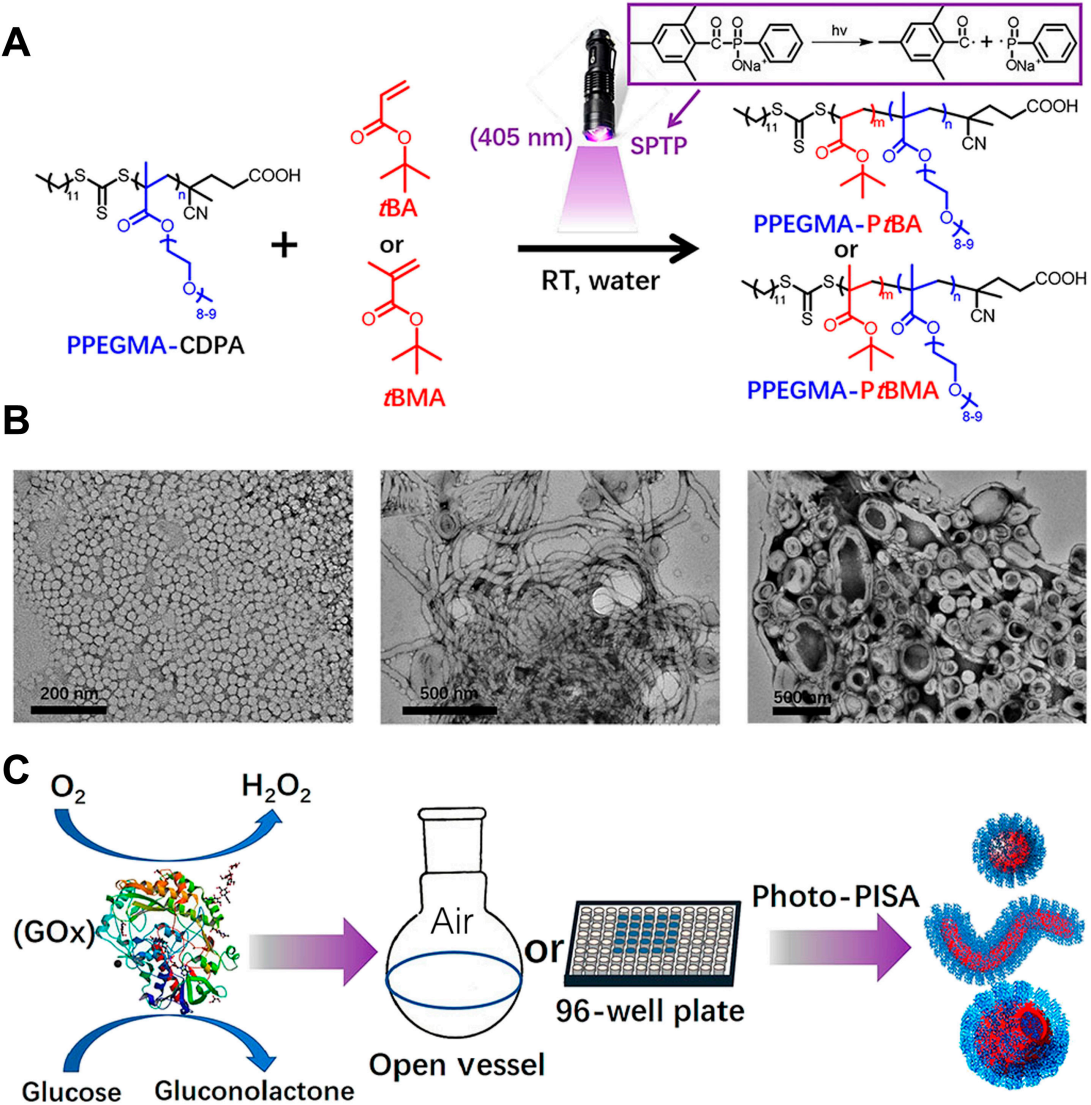
(A) Illustration for the preparation of PPEGMA-P*t*BA or PPEGMA-P*t*BMA diblock copolymers by photo-RAFT polymerization under ambient reaction condition. (B) Representative TEM images of spherical micelles composed of PPEGMA_6_-P*t*BMA_50_ and higher-order morphologies composed of PPEGMA_6_-P*t*BAn. (C) Schematic diagram of photo-PISA conducted in open vessel or well plate in the presence of GOx. Reproduced with permission from [72]. Copyright 2019, American Chemical Society.

Despite the successful preparation of polymeric NPs by UV-initiation PISA, the photolysis of the RAFT agent and loss of polymerization control by UV light are still the main problem. Therefore, visible light initiation is gradually becoming the new trend of photoinitiation. During the last decade, some research groups have reported visible light-initiated RAFT-PISA [72–80]. For instance, Zhang and coworkers reported the preparation of worms and vesicles by RAFT-mediated emulsion polymerization under ambient temperature (Fig. 3A) [60]. Specially, *tert*-butyl methacrylate (*t*BMA) and *tert*-butyl acrylate (*t*BA) were used as core-forming monomers. Compared with *t*BMA, *t*BA tended to induce the evolution of morphology, which may be caused by the lower glass transition temperature (*T*_g_) and weaker hydrophobicity (Fig. 3B). Furthermore, to overcome the quench effect of oxygen, glucose oxidase (GOx) and glucose were used, resulting in the success of RAFT-PISA in open vessels (Fig. 3C).

Photo-initiated RAFT-PISA is promising for the incorporation of bioactive materials into NPs because of its mild reaction condition. Some research work had successfully synthesized polymeric NPs with hydrophilic shell consisting of poly(amino acid)-based materials or polysaccharides [81–83]. For example, Gianneschi and coworkers fabricated proapoptotic peptide brush polymeric NPs by incorporating the proapoptotic “KLA” peptides, which could induce the apoptosis of cancer cells by disrupting the mitochondrial membrane [83]. Notably, the higher graft density of the KLA peptide in the obtained polymeric NPs had better anticancer efficiency according to the comparative experiment. Similarly, polysarcosine and polysaccharide have also been used as the hydrophilic shell for photo-PISA to improve biocompatibility and biodegradability [81,82].

Furthermore, photo-initiated RAFT-PISA can also be used as a robust technique to fabricate Janus inorganic/polymeric hybrid NPs. Janus gold NPs/block copolymers (Au@BCPs) have attracted much attention owing to their distinct physicochemical properties. However, the high reaction temperature of conventional thermal-initiated RAFT-PISA usually leads to AuNP aggregation. Therefore, photo-initiated RAFT PISA can be used as an alternative method to prepare Janus Au@BCP without high temperature. For this purpose, the P4VP-CTA end-capped with trithiocarbonate group was first decorated on the surface of AuNPs [84]. During the PISA process, by adjusting the ratio of monomer to CTA and reaction time, the size and morphology of Janus Au@BCPs could be precisely controlled.

Besides, continuous flow reactor has recently been designed to prepare polymeric NPs massively [85]. The morphology can be precisely tuned by adjusting the light intensity, solid content, and residence time. Notably, the continuous process can produce 60 g of polymeric NPs in 24 h, which has great advantages over the batch reactions. More recently, GOx and glucose were added into the reaction mixture to achieve photo-PISA without fussy degassing [86]. Through this strategy, polymeric NPs can be prepared on a large scale for biomedical application without batch-to-batch difference.

In general, compared with thermal-initiated RAFT-PISA, photo-initiated RAFT-PISA has more potential in constructing polymeric NPs when it involves the encapsulation of active molecules, which is attributed to the mild reaction of photo-PSIA [87]. According to the published literature, photo-PISA was preferred to build biological platforms.

#### Enzyme-initiated RAFT-PISA

Despite the fact that photo-initiated RAFT polymerization can be performed on a mild condition, some drawbacks still exist. For instance, the limited depth of light penetration can cause the incomplete monomer conversion. In addition, photosensitive materials are not suitable for this reaction system.

**Fig. 4.**
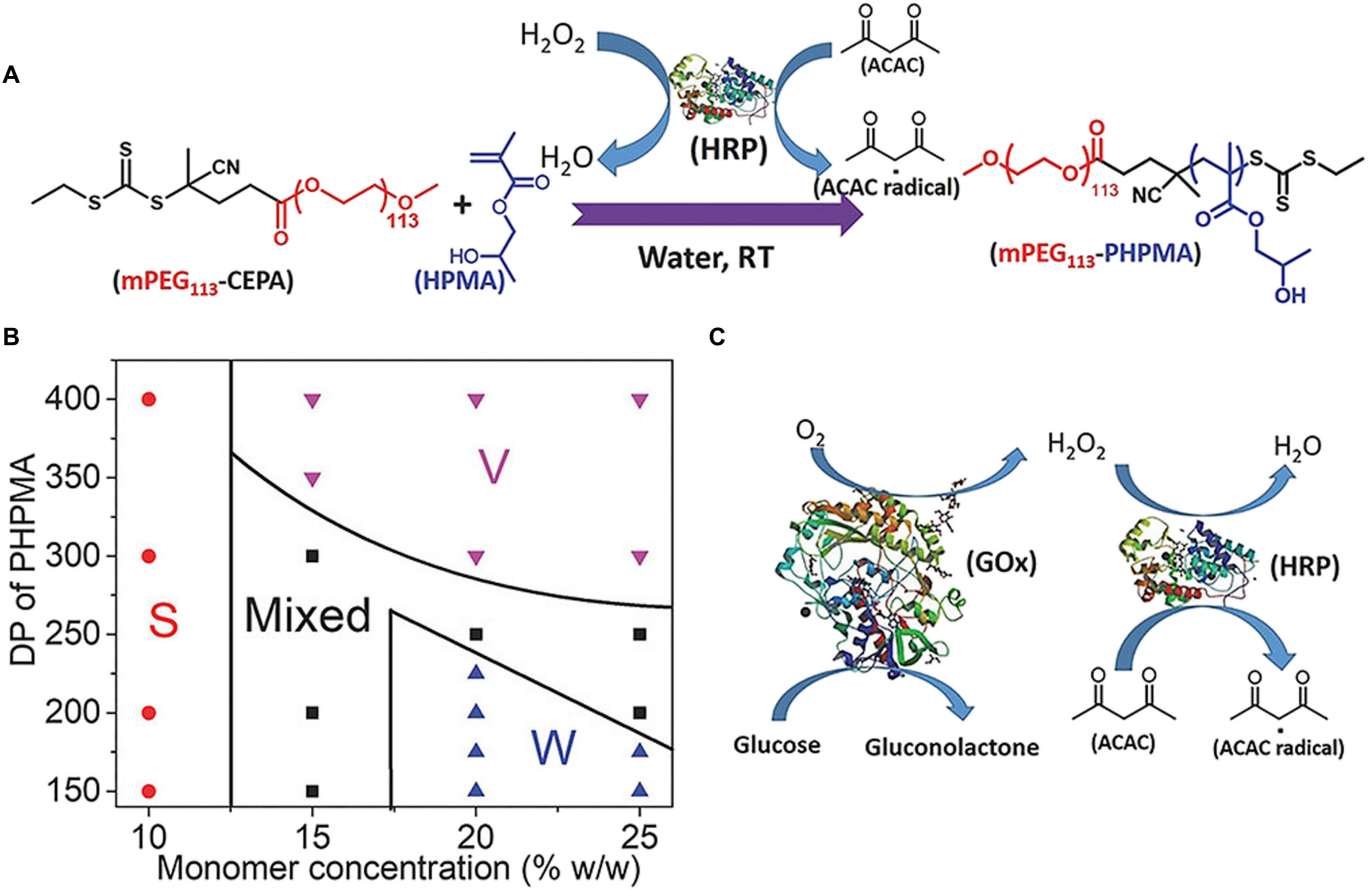
(A) Amphiphilic polymer mPEG_113_-PHPMA prepared via enzyme-RAFT in water. (B) Phase diagram where the DP of PHPMA block and the solid content varied. (C) Schematic illustration of GOx-HRP cascade reaction. Reproduced with permission from [94]. Copyright 2018, Wiley.

To date, enzyme-initiated RAFT polymerization has been successfully conducted [88–91]. These works realized a mild and efficient PISA by utilization of the horseradish peroxidase/hydrogen peroxide/acetylacetone (HRP/H_2_O_2_/ACAC) ternary initiating system. In addition, GOx was also exploited to produce free radicals in the presence of oxygen [92,93]. For example, Tan and coworkers first introduced enzyme initiation into RAFT-PISA [94]. In this case, mPEG113-4-cyano-4-(ethylthiocarbonothioylthio) pentanoic acid (mPEG_113_-CEPA) was used as macro-CTA for the polymerization of 2-hydroxypropyl methacrylate (HPMA) units (Fig. 4A). By adjusting the solid content and DP of the HPMA block, various morphologies can be obtained, and detailed phase diagram was drawn (Fig. 4B). During the reaction process, free radicals were generated through the oxidation of ACAC by hydrogen peroxide (H_2_O_2_) in the presence of HRP. To avoid the influence of oxygen, GOx was used as catalyst to transform oxygen into H_2_O_2_ in the presence of glucose (Fig. 4C). In this way, complicated freeze–pump–thaw cycles were not necessary. Notably, the vesicles had the ability to load both biomacromolecules and inorganic materials. As characterized by TEM, the SiO_2_ NPs and bovine serum albumin (BSA) could be loaded in the cavities of polymeric vesicles, and the BSA had little effect on the formation of vesicular morphology.

As known, the epoxy group is unstable under high temperature (e.g., 80 °C). Therefore, traditional thermal-initiated RAFT-PISA is not suitable for preparing epoxy-functionalized NPs. Impressively, epoxy-functionalized polymeric NPs can be prepared by photo-initiated RAFT-PISA [95]. Besides, enzyme-initiated seeded RAFT-PISA can also be used to prepare epoxy-functionalized triblock copolymer vesicles [89]. In this case, the prepared poly(glycerol monomethacrylate)_46_-PHPMA_300_ vesicles were used as macro-CTA for the polymerization of glycidyl methacrylate (GlyMA) monomers, and the surface of vesicles became rough by increasing the DP of PGlyMA block.

In a word, enzyme-initiated RAFT-PISA is a mild strategy to fabricate polymeric nano-objects on a large scale. Compared with photo-initiated PISA, it expands the scope of monomers, which can be involved in the mild PISA process. However, the current enzyme-initiated PISA experiments were carried out in aqueous solution. Therefore, exploring more types of enzymes suitable for PISA and improving the stability of enzyme in organic solvent are worthy of further study.

#### Ultrasound-initiated RAFT-PISA

Compared with above RAFT-PISA, ultrasound-initiated RAFT-PISA does not need traditional initiators [96]. Free radicals for ultrasound-initiated RAFT-PISA are generated by high-frequency ultrasound. Different from photo-initiated RAFT-PISA, the free radicals dispersed evenly throughout the reaction system, avoiding the problem of uneven distribution of free radical concentration. In 2018, the first ultrasound-initiated RAFT-PISA was successfully performed in water by using high-frequency ultrasound (490 kHz) with monomer conversion nearly 100% in 60 min [97]. The obtained poly(poly(ethylene glycol) methyl ether acrylate)-poly(*N*-isopropylacrylamide*-co-N*,*N*′-methylenebis(acrylamide)) (PPEGA-P(NIPAM*-co-*MBA)) nanogels had the lower critical solution temperature (LCST)-type thermosensitive characteristic. Because the LCST of PNIPAM block was 32 °C, when temperature was increased from 25 to 45 °C, the size and polydispersity index (PDI) of polymeric NPs decreased, which was caused by the collapse of P(NIPAM*-co-*MBA) block. Notably, ultrasound wave-initiated RAFT-PISA can be used for expanding the scope of functional materials.

**Fig. 5.**
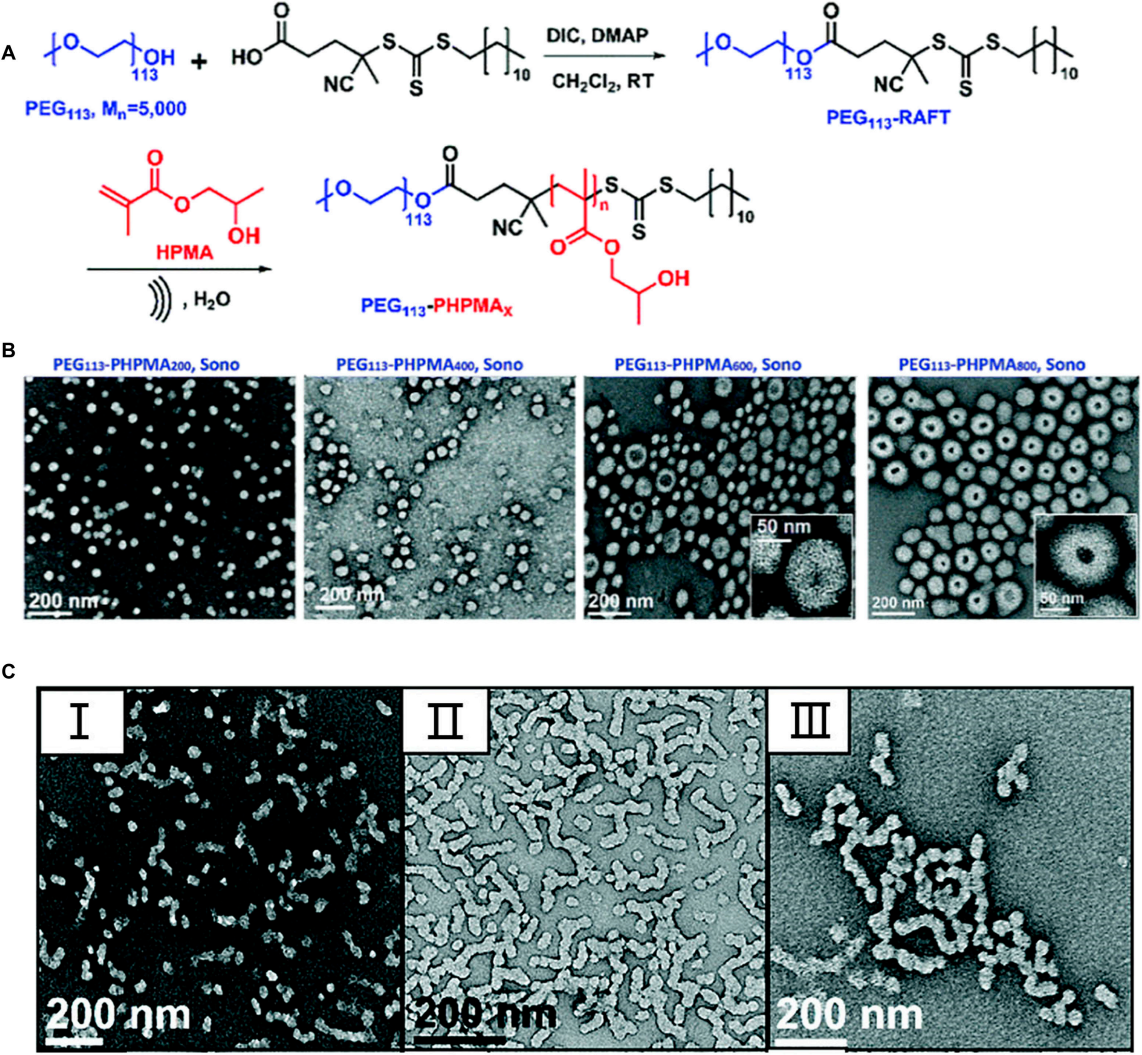
(A) Synthetic process of diblock copolymers PEG_113_-PHPMA*_n_* via ultrasound wave-initiated RAFT-PISA. (B) TEM images of PEG_113_-PHPMA*_n_* NPs with various morphologies prepared at 10% (w/w) solid content. (C) Representative TEM images of worms prepared by Sono-PISA using CCL strategy. Reproduced with permission from [98]. Copyright 2020, Royal Society of Chemistry.

For another, Thang *et al.* adopted the ultrasound (990 kHz) to prepare polymeric NPs with various morphologies (Fig. 5A) [98]. The work first prepared vesicle morphology by Sono-RAFT-PISA (Fig. 5B). For comparison, thermal-initiated RAFT-PISA was also investigated. Compared to thermal initiation, polymeric vesicles were formed at shorter PHPMA length by ultrasound-initiated RAFT-PISA. In addition, the size of obtained vesicles prepared by ultrasound-initiated RAFT-PISA was much smaller (126.2 nm versus 599.2 nm), and the PDI was also narrower (0.02 versus 0.23). However, worm structures were difficult to achieve due to high ultrasonic frequency. To overcome the influence of acoustic streaming effect, the core cross-linking (CCL) strategy was used as a solution to prepare wormlike nano-assemblies (Fig. 5C).

Ultrasound-initiated RAFT-PISA is emerging as a promising technique to fabricate NPs on a large scale. More importantly, the water in the system is used not only as a solvent but also as an initiation source. Therefore, no external initiators and additives are needed, which makes the ultrasound-initiated RAFT-PISA technique quite “green.” Compared with thermal initiation and photo-initiation, ultrasound-initiated RAFT-PISA does not suffer from uneven heating and insufficient depth of light penetration. However, the effect of high ultrasonic frequency on the morphology of nano-assemblies during the PISA process still needs further studies.

### ATRP-PISA

ATRP is one of the most well-developed controlled polymerization techniques [99,100]. Compared with other polymerization techniques, the unique characteristic of ATRP is the requirement of transition metal catalyst. In addition, the polymerization condition is mild without high temperature and pressure. ATRP can be conducted in various single or mixed solvents, such as MeOH, water/MeOH, and supercritical carbon dioxide (scCO_2_) [101]. However, according to amounts of the published work, ATRP-PISA does not seem to be a good PISA formulation, mainly because transition metal complexes can bring potential toxicity into polymeric NPs and unpleasant control. The first ATRP-PISA work was reported by Pan and coworkers in 2007 [102]. What is more, after the reaction, the remaining metal catalysts in the nanostructures are harmful for use, especially in biomedical application [103]. How to improve and optimize the reaction condition of ATRP is the key issue in recent years.

**Fig. 6.**
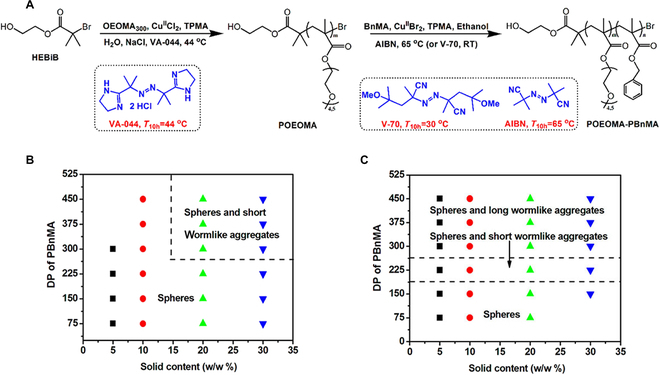
(A) Diblock copolymer POEOMA_50_-PBnMA*_x_* prepared by ICAR ATRP with relevant initiators at different temperature. (B) Phase diagram of POEOMA_50_-PBnMA*_x_* self-assemblies at ambient temperature. (C) Phase diagram of POEOMA_50_-PBnMA*_x_* self-assemblies at 65 °C. Reproduced with permission from [104]. Copyright 2016, American Chemical Society.

Notably, initiators for continuous activator regeneration atom transfer radical polymerization (ICAR ATRP) is a powerful method to improve the reaction process by lowering the concentration of metal catalyst. In 2016, ICAR ATRP-PISA was first used to prepare poly(oligo(ethylene oxide) methyl ether methacrylate)-*b*-poly(benzyl methacrylate) (POEOMA*-b-*PBnMA) with the parts per million (ppm) concentration of copper catalyst (Fig. 6A) [104]. Here, Matyjaszewski and coworkers used the CuBr_2_/tris(pyridine-2-ylmethyl)amine (TPMA) complex as catalyst at RT or at 65 °C with various radical initiators. In detail, the research studied the effect of the catalyst concentration, solid content, and temperature on the self-assembly behavior. Notably, the glass transition temperature (*T*_g_) of the core-forming block was 54 °C, which was between RT and 65 °C. Therefore, various assembly behaviors were observed. When at ambient temperature, large spheres were observed as the main morphology. As the solid content and the DP of PBnMA increased, a fractal-type connected-bead structure was finally obtained (Fig. 6B). However, when the temperature was 65 °C, the morphology transition from spheres to worms also appeared at low solid content (10%, w/w) (Fig. 6C). The reduced catalyst concentration in ATRP-PISA can expand the scope of the relevant applications. More recently, Wang’s group explored the morphological evolution of NPs using mixed POEOMA_24_ and POEOMA_78_ as macroinitiator [105]. Other polymers such as methoxy-poly(ethylene oxide)-polyacrylonitrile (mPEO_113_-PAN) was successfully synthesized via ICAR ATRP [106]. Spherical and wormlike NPs could be obtained through self-assembly. The reduced catalyst concentration in ATRP-PISA can expand the scope of the relevant applications.

In addition, activators regenerated by electron transfer atom transfer radical polymerization (ARGET ATRP) is another method to achieve ATRP process with lower concentration of catalyst (ppm) [107,108]. In such a process, high-valent transition metal complexes were used as catalysts and excess reducing agents were added intermittently to continuously reduce the high-valent transition metals to low-valent oxides to effectively reduce the amount of catalyst. For instance, Matyjaszewski and coworkers reported the fabrication of crossed-linked NPs in ethanol by ARGET ATRP-PISA [109]. Here, POEOMA with a terminal bromide was used as macro-CTA to extend with glycidyl methacrylate (GMA) or BnMA monomers. The different conversion rate between double bond and oxirane ring in GMA guaranteed the PISA process and the subsequent in situ cross-link of NPs.

Organic–inorganic materials have been widely applied in catalyst, imaging, and nanomedical fields. To realize practical application of ATRP-PISA, a novel NP consisting of POEOMA*-b-*poly (2-(perfluorohexyl)ethyl methacrylate (PHFEMA)*-co-*GMA) had been prepared by ATRP-PISA [110]. Afterward, mercapto-succinic acid was used to react with the epoxy group on the GMA, and the carboxyl groups were successfully introduced into the core of polymeric NPs. Subsequently, the nano-objects can be used as template to prepare Fe_3_O_4_@POEOMA organic–inorganic NPs. As characterized by TEM, dynamic light scattering (DLS), and thermogravimetric analysis (TGA), Fe_3_O_4_@POEOMA with an average diameter of about 150 nm was prepared. In addition, the diameter could be modulated by varying the packing parameter.

As known, the solvent, solid content, target DP, and temperature are key factors on the morphology of the nano-assemblies prepared by PISA. Whether different PISA formulations can fabricate different morphologies with the same BCP composition is a question worth exploring. In 2017, Zhang and coworkers reported the fabrication of diblock copolymer poly(2-hydroxypropyl methacrylate*-b-*benzyl methacrylate) (PHPMA*-b-*PBzMA) nano-assemblies to make a comparison between ATRP and RAFT [111]. The results indicated that both 2 robust polymerization techniques had good control on the MW and MW distribution. Notably, the ATRP dispersion polymerization was faster than the RAFT dispersion polymerization. In addition, nano-objects prepared by ICAR ATRP-PISA were larger than that prepared by RAFT polymerization.

More recently, the same group made another comparison between ATRP-PISA and RAFT-PISA [112]. The PEG*-b-*PS nano-assemblies were prepared by ATRP-PISA and RAFT-PISA. Although the targeted DP of PS was the same, the high copolymer dispersity existing in ATRP-PISA led to various morphologies. In addition, the concentration of Cu salt on the effect of morphology was explored. With the increase of Cu salt concentration, the flower-like structure was formed. As a robust PISA formulation, ATRP-PISA can be used to prepare functional NPs on a large scale. Compared to RAFT-PISA, ATRP-PISA is usually performed at RT and does not involve the sulfur-containing structure.

### ROP-PISA

As mentioned above, nano-assemblies including spheres, worms, and vesicles can be prepared by controlled radical polymerization technique-mediated PISA. Nevertheless, the suitable monomers are mainly methacrylates and vinyls. However, the obtained NPs constructed by these PISA methods are usually not biodegradable. In this section, ROP-PISA including ring-opening metathesis-mediated PISA (ROMP-PISA), ROP of *N*-carboxyanhydride-induced self-assembly (NCA-PISA), and radical ROP-induced self-assembly (rROP-PISA) will be elaborated carefully.

#### ROMP-PISA

**Fig. 7.**
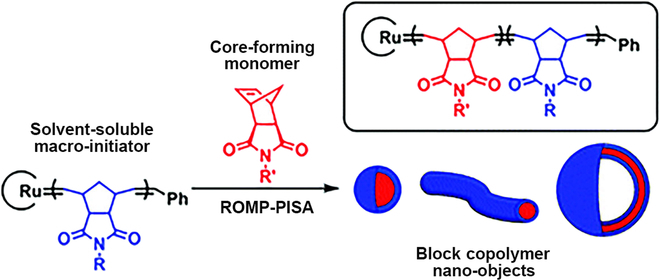
Schematic illustration of various structures prepared by ROMP-PISA. Reproduced with permission from [113]. Copyright 2019, Royal Society of Chemistry.

ROMP is a nonradical method to achieve the polymerization process with Ru-based catalysts under mild reaction conditions (Fig. 7). Cyclic monomers are used in the ROMP-PISA process to fabricate polynorbornene (PNB)-based NPs with various morphologies. In recent years, due to extraordinary functional group tolerance, ROMP-PISA is emerging as a robust approach to prepare functional NPs under mild condition [113].

For example, Xie and coworkers first reported the facile preparation of functionalized polymeric nano-assemblies by the ROMP-PISA technique [114]. The homopolymer poly(2,3-dihydroxymethyl-5-norbornene) (PBNBE) was used to be chain-extended with 7-oxanorborn-5-ene-exo-exo2,3-dicarboxylic acid dimethyl ester (ONBDM) monomers and self-assembled into NPs in toluene. Later, the same group reported the preparation of triblock copolymer PBNBE*-b-*poly(exo-*N*-(cinnamoyloxyethyl)-7-oxanorborn-5-ene-2,3-dicarboximide) (PCONBI)*-b-*PONBDM with UV-crosslink core [115]. Through cross-linking strategy, fabricated NPs retained the original morphology in good solvent as CHCl_3_, which indicated that the stability of obtained NPs had been improved greatly. Further, relevant works where higher-order morphologies and functional NPs were generated were reported by other groups [116,117].

Further, more and more functional monomers have been used in ROMP-PISA to achieve desired applications in recent years. Char and coworkers first reported the sulfur-rich polymeric NPs constructed by ROMP-PISA [118]. When increasing the ratio of the sulfur-containing monomer during the PISA process, the refractive index changed in a controlled manner, which could be used for monitoring the reaction process. In addition, enzyme-responsive polymeric NPs were prepared under open-to-air condition [119]. First, l-amino acid-based norbornene dicarboximide peptide substrate with sequence GPLGLAGGWGERDGS was chose to synthesize hydrophilic block, which was then chain-extended with quaternary aminebased phenyl norbornene dicarboximide to self-assemble into spherical micelles. The recognition sequence PLGLAG existing in the hydrophilic shell was the substrate of enzyme thermolysin. Therefore, the obtained NPs would undergo phase transitions when exposed to the proteolytic thermolysin.

However, a major obstacle to conduct aqueous ROMP-PISA is the poor solubility of Grubbs catalyst in water. One solution to make catalyst water-soluble is to modify it by introducing hydrophilic chain segment [120,121]. For this purpose, a single cationic quaternary amine Hoveyda–Grubbs second-generation initiator was used in the ROMP-PISA to build well-defined NPs and detailed phase diagram was produced [120]. Recently, highly hydrophilic Grubbs catalyst was developed to obtain polymers with excellent control on MW and dispersity [122]. First, a short hydrophilic chain was polymerized in tetrahydrofuran (THF) in the presence of G3. Afterward, the obtained macroinitiator could be chain-extended with second hydrophobic monomers. During ROMP-PISA, various morphologies were observed, such as spheres, worms, and vesicles.

#### ROP-PISA of NCA monomers

**Fig. 8.**
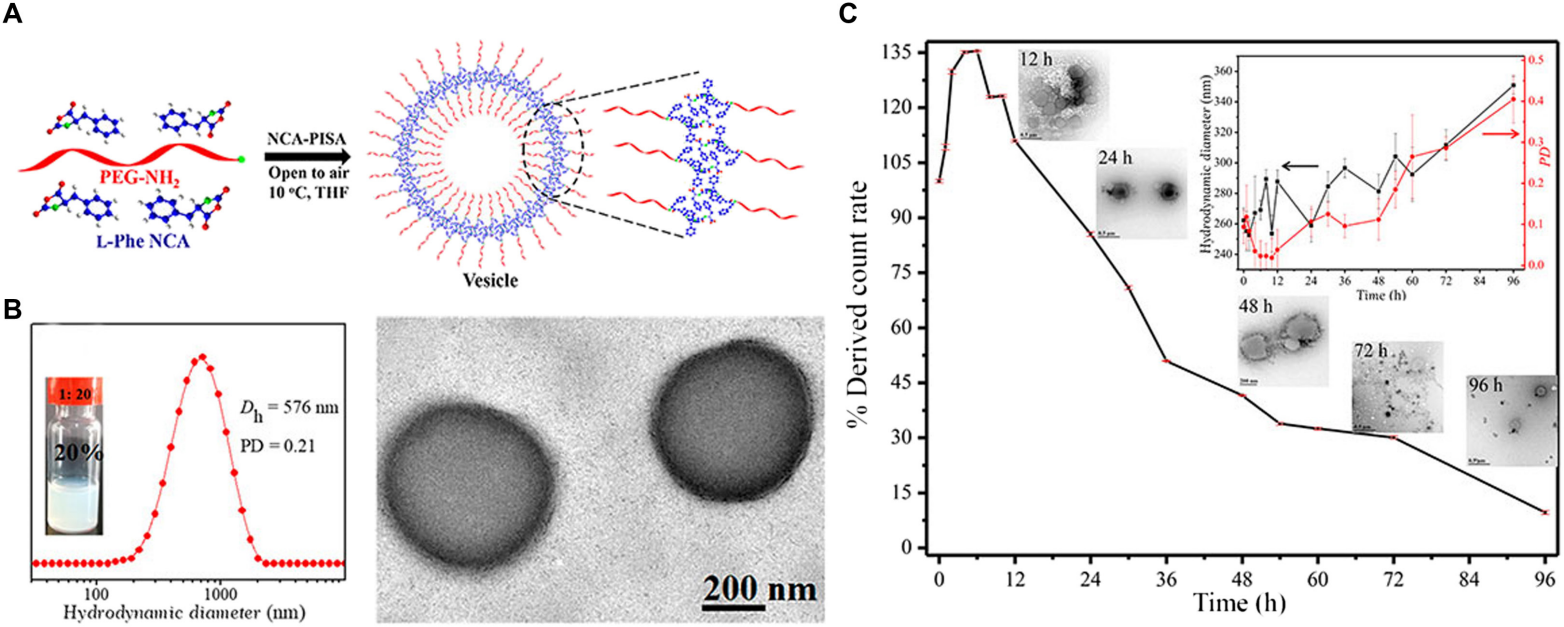
(A) Schematic illustration of biodegradable NPs prepared by NCA-PISA at 10 °C in tetrahydrofuran. (B) DLS characterization and TEM images of vesicles when the DP of core-forming block was 20 and the solid content was 20% (w/w), respectively. (C) Enzymatic degradation study of polymer vesicles in vitro. Reproduced with permission from [125]. Copyright 2019, American Chemical Society.

Polypeptide-based materials have been always attractive due to their distinct advantages including excellent biocompatibility and biodegradability [123,124]. Traditional self-assembly of polypeptides is generally conducted in a selective solvent, which is inefficient and time-consuming. A facile one-pot preparation strategy of polypeptide NPs was first reported by Du and coworkers in 2019 [125]. Here, PEG_45_-NH_2_ macroinitiator was used to polymerize with l-phenylalanine NCAs (l-Phe NCAs) in anhydrous THF (Fig. 8A). Compared with the above PISA formulations, this ROP-PISA was conducted on the highly mild condition (10 °C) and the whole process did not need additional species. The hydrophilic/hydrophobic ratio and solid content could be finely regulated to achieve vesical morphology (Fig. 8B). Furthermore, the in vitro enzymatic degradation experiments showed that most vesicles were degraded in 96 h when treated with trypsin (Fig. 8C). Reactive oxygen species (ROS)-responsive methionine NCA monomer was also applied as hydrophobic block to fabricate polymeric NPs under high solid content (up to 45%) [126]. The obtained polymeric NPs were incubated in different concentrations of H_2_O_2_ (e.g., 0.1, 1, and 10 mM) to investigate their oxidative degradation behavior. Notably, these vesicles could be quickly degraded under various H_2_O_2_ concentrations, which had great potential as intelligent nanocarriers. The novel NCA-PISA brings great potential for biomedical applications including drug delivery and bioimaging.

More importantly, aqueous ROP-PISA of NCAs, which was first reported by Bonduelle’s group in 2019, is a big breakthrough because NCA monomers are believed to be moisture-labile [127]. To be specific, sodium bicarbonate aqueous solution (pH 8.5, 50 mM) was used to prevent the hydrolysis of NCAs and promoted the ROP-PISA process. PEG5k-NH_2_ was used as the macroinitiator to be chain-extended with γ-benzyl-L-glutamate *N*-carboxyanhydrides (BLG-NCAs) at 4 °C. The aqueous NCA-PISA not only fabricated some conventional morphologies including spheres and worms but also obtained homogeneous needle-like structures. It was envisioned that this ROP-PISA process was able to use different macroinitiators and NCA monomers. Notably, owing to the unique feature of polypeptides (e.g., secondary structure), NCA-PISA is different from the above PISA formulations, which is also worthy of further exploration.

Very recently, the same group reported the effect of polypeptides’ secondary structure on the morphologies of formed NPs in 2021 [128]. Two different monomers, i.e., BLG-NCA and Leu-NCA, that possessed α-helix and β-sheet conformation, respectively, were used to investigate the influence of secondary structure on ROP-PISA. The results demonstrated that the Leu-NCA monomers enhanced the anisotropic character of the resulting self-assemblies. In fact, compared with α-helical polypeptides, β-sheet polypeptides tended to form long rods with high aspect ratio. As a novel PISA technique, more and more research work is worth doing to explore the formation mechanism of NPs.

More recently, Du and coworkers carefully studied the effect of monomer chirality in chirality-controlled PISA by adjusting the chiral molar ratio of the Phe NCAs [129]. Various chiral composition also leads to different α-helix fraction and β-sheet fraction. When β-sheet fraction increased to 50%, the uniform PISA behavior disappeared, which meant that the β-sheet structure could weaken assembly behavior. Notably, in addition to morphological control, by increasing of D-Phe fraction, the biodegradability of obtained NPs decreased. Adjustable biodegradation rate can be applied to control the drug release rate, thus having significant potential for biological applications.

#### rROP-PISA

Improving the biodegradability of polymers is of great significance for bioapplications. Radical ROP is a method in which degradable groups (e.g., ester and thioester) can be introduced into the main chain of the polymer structure. Recently, Nicolas and coworkers first reported the rROP-PISA based on cyclic ketene acetals (CKAs) in heptane, such as 2-methylene-4phenyl-1,3-dioxolane (MPDL) and 5,6-benzo-2methylene-1,3-dioxepane (BMDO) [130]. As expected, the introduction of ester groups greatly improved the degradation property, which was verified by degradation test between the MPDL-free poly(lauryl methacrylate)*-b-*PBzMA (PLMA*-b-*PBzMA) copolymer and PLMA*-b-*P(BzMA*-co-*MPDL) copolymers. Notably, by increasing the ratio of CKA, the better degradation could be achieved.

More recently, aqueous suspensions of degradable copolymer NPs were prepared by the 2-step rROP-PISA process [131]. First, poly[oligo(ethylene glycol) methyl ether methacrylate] (POEGMA) was chosen as solvophilic block to be chain-extended with the 3 main CKA monomers [e.g., MPDL, BMDO, and 2-methylene-1,3-dioxepane (MDO)] to in situ fabricate NPs in *N*,*N*-dimethylformamide (DMF). Then, the suspensions of NPs were dialyzed against water. To be specific, the POEGMA chain preserved the stability of NPs when the formation was transferred from DMF to water. Notably, the longer POEGMA block provided better stability of NPs especially for high CKA contents. All these CKA-containing NPs exhibited quick hydrolytic degradation under accelerated conditions (2.5 wt% KOH), proving that degradable vinyl polymeric NPs have great potential in bioapplications (Table 1).

## Biomedical Applications

Nowadays, design and construction of biomedical nanoplatforms has gained great significance especially in the field of human healthcare. The stability of therapeutic agents can be greatly improved by encapsulation in NPs. Furthermore, by incorporation of targeting moieties, these NPs can efficiently target the disease sites. In recent years, PISA has been proved to be a robust technique to prepare functional NPs. Therefore, fabricating biomedical platform by PISA can not only achieve greater value of PISA but also improve the efficiency of the preparation [32,34,132]. So far, a range of polymeric NPs have been fabricated by PISA strategy for biomedical applications. In the following section, the efficient encapsulation and controlled release of different cargoes including imaging agents, anticancer drugs, and enzymes will be demonstrated in detail (Table 2).

### Bioimaging

The premise of precise treatment on diseases is able to precisely assess early diagnosis. Therefore, bioimaging has great significance on medical diagnosis and subsequent therapy. Current biological imaging techniques consisted of magnetic resonance imaging (MRI), computed tomography (CT), fluorescence imaging (FI), etc. [133]. Imaging agents are generally requested for precise detection of diseases. However, these small imaging agents usually have poor biodistribution and unsatisfied signal-to-noise ratio. Notably, by encapsulation of these imaging agents in polymeric NPs, the above issues can be greatly alleviated.

#### CT imaging

**Fig. 9.**
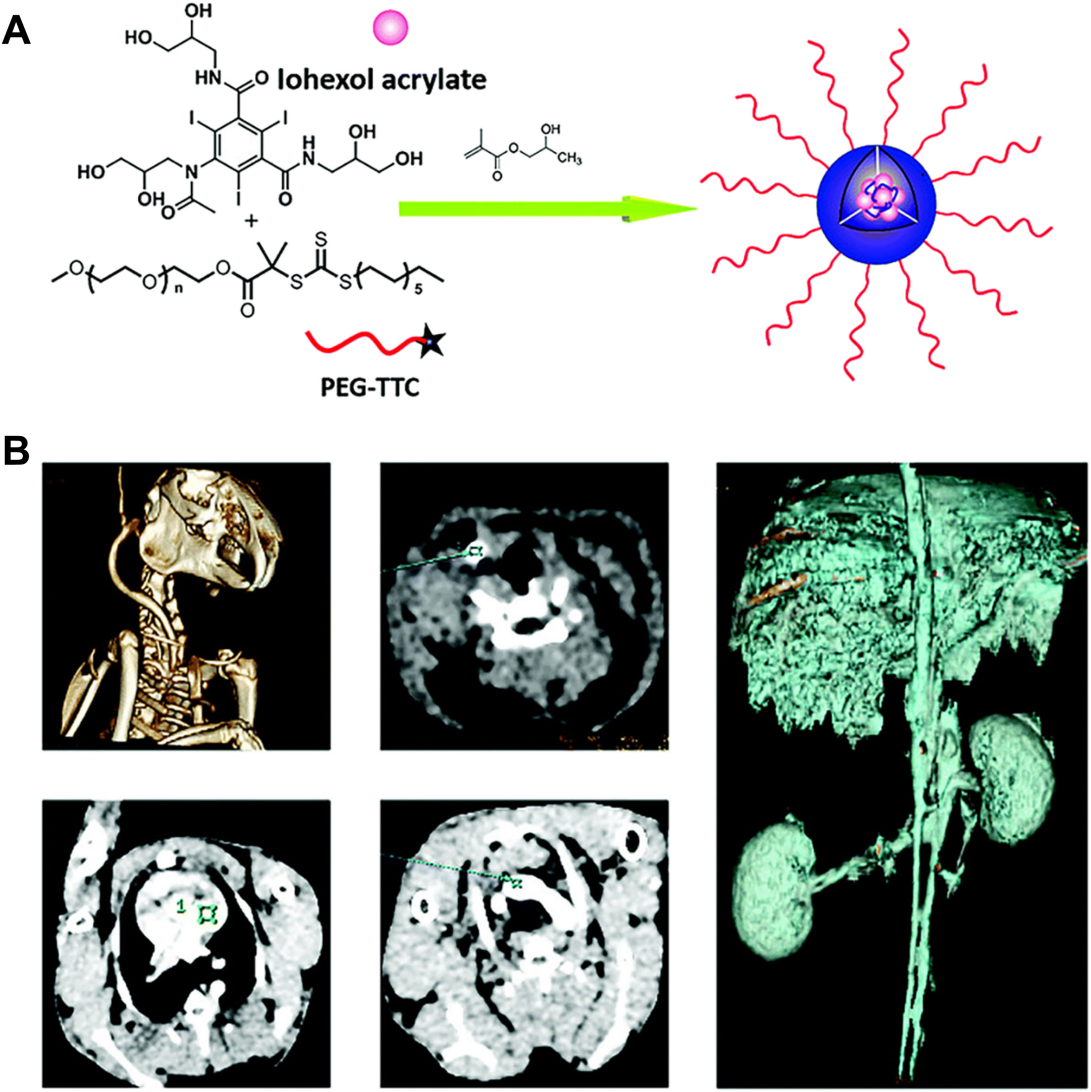
(A) Schematic illustration of INPs prepared by RAFT-PISA. (B) The CT images of blood vessels (e.g., jugular vein, left ventricular, and aortic arch) and organs (e.g., liver and kidney). Reproduced with permission from [136]. Copyright 2018, Royal Society of Chemistry.

As a common clinical imaging technique, CT has been widely used owing to its high efficiency [134,135]. However, the conventional contrast agents (e.g., iohexol) are easy to be cleared quickly and distribute highly in normal tissues and organs, and iodinated NPs can overcome the above limitations. The most iodinated NPs prepared by traditional self-assembly method cannot meet the requirements of clinical imaging owing to the complicated and low loading efficiency of iodine. To overcome above limitations, iohexol NPs (INPs) have been successfully fabricated by PISA in high concentration (up to 120 mg ml^-1^) [136]. Here, the macro-CTA PEG5k-*S*-1-dodecyl-*S*ʹ-(α,α′-dimethyl-α′′-acetic acid) trithiocarbonate (PEG5k-TTC) was chain-extended with hydrophobic monomer HPMA to fabricate polymeric NPs (Fig. 9A). Then, iohexol acrylate was used as crosslinking agent to achieve efficient loading. Notably, the loading efficiency of iohexol in these NPs reached 36.5%, endowing these INPs great potential in CT as contrast agent. The in vivo pharmacokinetic studies showed that INPs had a longer half-life compared with free iohexol and a higher-level CT signal. Notably, the INPs had low accumulation in liver and renal tissues, resulting in low biological toxicity and extended retention time. As expected, the novel INPs had a significant accumulation in tumor rather than normal tissues, which greatly improved the sensitivity of CT imaging in cancer diagnosis. In addition, according to the 3-(4,5)-dimethylthiahiazo (-z-y1)-2,5-di-phenytetrazoliumromide (MTT) assay in vitro, the viabilities of cells incubated with INPs were all over 90%, which showed high biocompatibility of INPs. Finally, by performing CT scans on the animals treated with INPs, main organs (e.g., liver and kidney) and representative blood vessels (e.g., renal vein, renal artery, aorta jugular vein, and the left ventricle) could be imaged clearly (Fig. 9B). These above results proved the INPs could effectively improve diagnostic accuracy and reduce biotoxicity.

#### MRI imaging

In addition to CT imaging, MRI as another conventional imaging technique also requires contrast agents to enhance the image quality, because it is easily affected by the high background signal from water. To improve the imaging quality, a strategy designed to minimize an overwhelming background signal and largely prepare polymeric NPs had been reported [137]. Briefly, ^19^F-containing poly(oligo(ethylene glycol) methyl ether methacrylate-*co*-2,2,2-trifluoroethyl acrylate) [poly(OEGA*-co-*TFEA)] was used as macro-CTA to fabricate polymeric NPs with various morphologies. As expected, all fluorinated NPs had detectable ^19^F MRI signal, in which the spherical NPs with the highest fluorine content (0.26 wt%) had the highest ^19^F MRI signal-to-noise ratio (8.09 × 10^6^ ms). Notably, the ^19^F nuclear magnetic resonance (NMR) relaxation time *T*_1_ and *T*_2_ values of various morphological NPs were approximate, indicating that the dynamics of the corona of different NPs were identical. In addition, the effect of the morphology on cell uptake was investigated. Consistent with previous studies, the wormlike nanostructure with higher aspect ratio had the highest cell uptake efficiency. However, the vesicle NPs were difficult to be uptaken by cells. The cell uptake result was also confirmed by confocal laser scanning microscopy (CLSM). All these results showed the fluorinated NPs with wormlike morphology had the potential for in vivo cell tracking and cell imaging. Furthermore, more fluorinated NPs can be constructed by PISA to perform bioimaging and disease diagnosis.

**Fig. 10.**
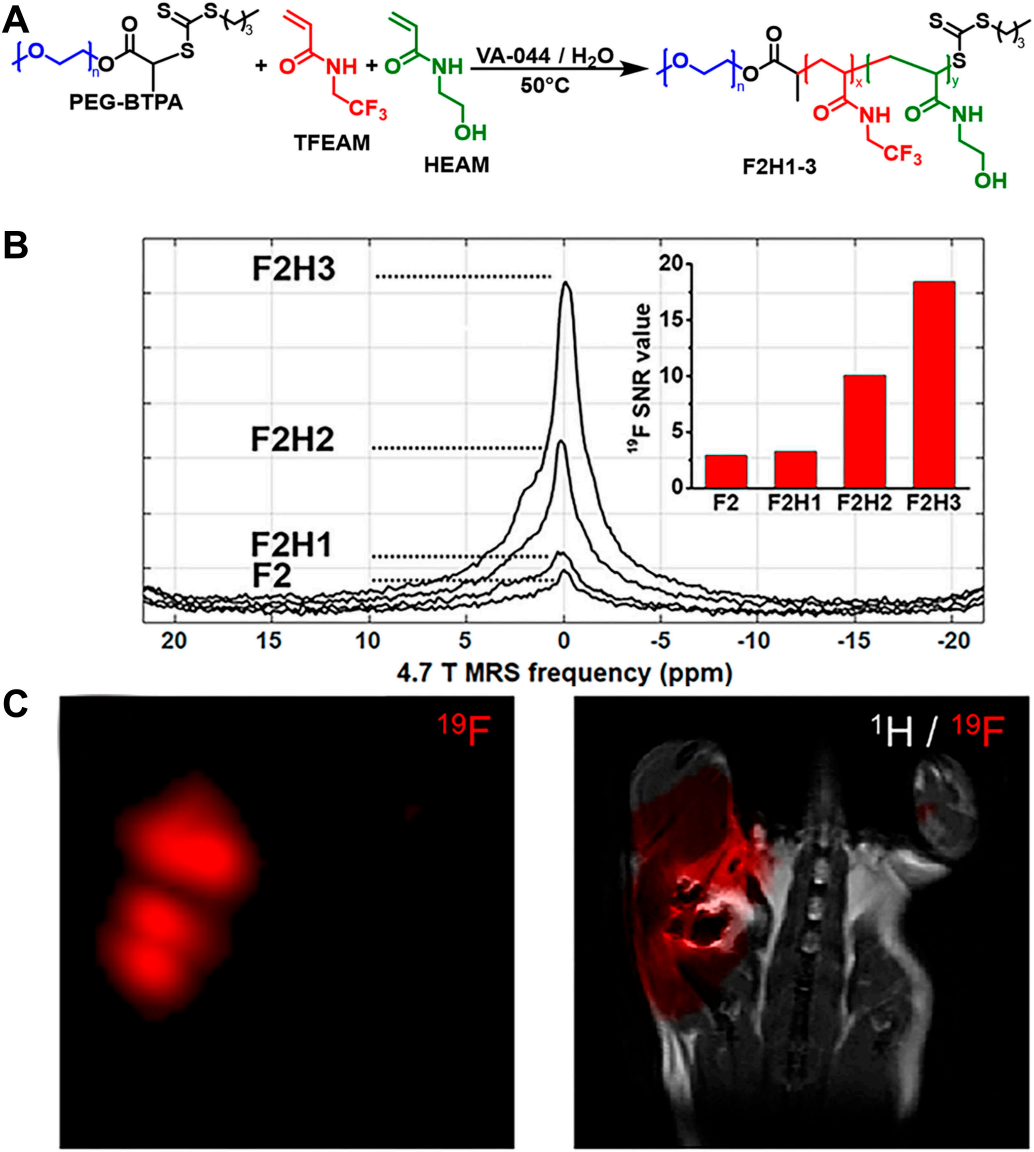
(A) Schematic illustration of PEG-*b*-(PTFEAM-stat-PHEAM) fluorinated NPs prepared by aqueous RAFT-PISA. (B) The ^19^F MR spectra (4.7 T) and the ^19^F MRI SNR values of various NPs. (C) The in vivo ^19^F MRS and MRI images of BALB/c mouse after the injection of F5H2 nanotracers. Reproduced with permission from [138]. Copyright 2022, American Chemical Society.

More recently, ^19^F MRI nanotracers were first fabricated by aqueous RAFT-PISA, using *N*-(2,2,2-trifluoroethyl)acrylamide (TFEAM) as core-forming monomers (Fig. 10A) [138]. However, the PEG-*b*-PTFEAM NPs did not have strong ^19^F NMR signal, which was attributed to the limited mobility of the core-forming blocks, further leading to the poor magnetic relaxation properties. Therefore, hydrophilic *N*-hydroxyethyl acrylamide (HEAM) monomer was used to improve chain hydration and mobility. With the increasing content of HEAM (up to 30%), the signal-to-noise ratios (SNRs) of the NP dispersions gradually increased (Fig. 10B). In addition, for further biomedical application, ensuring the stability of the micelles, PEG_91_-b-(PTFEAM_320_-stat-PHEAM_80_) (F5H2) was used as nanotracer. In in vivo imaging experiment, after the injection of F5H2 nanotracers into the right hind leg of mouse, obvious ^19^F MRI signals with excellent SNRs could be monitored, indicating that the novel fluorinated NPs constructed by PISA had great potential in biomedical imaging (Fig. 10C).

#### Fluorescence imaging

In addition, fluorescent materials have been developed and grown vigorously in recent years owing to the great potential in biomedical fields [139–141]. Some organic fluorescent molecules are limited in the bioapplication, ascribed to poor solubility in water. RAFT-PISA is a robust technique to prepare fluorescent NPs for bioimaging.

For instance, chalcone-containing NPs with uniform spherical morphology had been successfully prepared by using chalcone derivative terminated with ethylene group [142]. Interestingly, when the NPs were transferred from dioxane to water, the size shrank distinctively from ~200 nm to ~100 nm. Furthermore, the emission intensity significantly improved, which was contributed to the aggregation-induced emission feature of chalcone. The biocompatibility of the fluorescent NPs was characterized by CCK-8 kit assay. The cell still maintained high cellular activity (above 90%), although the concentration of NPs reached 40 μg/ml. Further, HeLa cells were cultivated with fluorescent NPs (10 μg/ml) for cell imaging. The obtained fluorescent images with high contrast confirmed the great potential of fluorescent NPs in cell imaging.

**Fig. 11.**
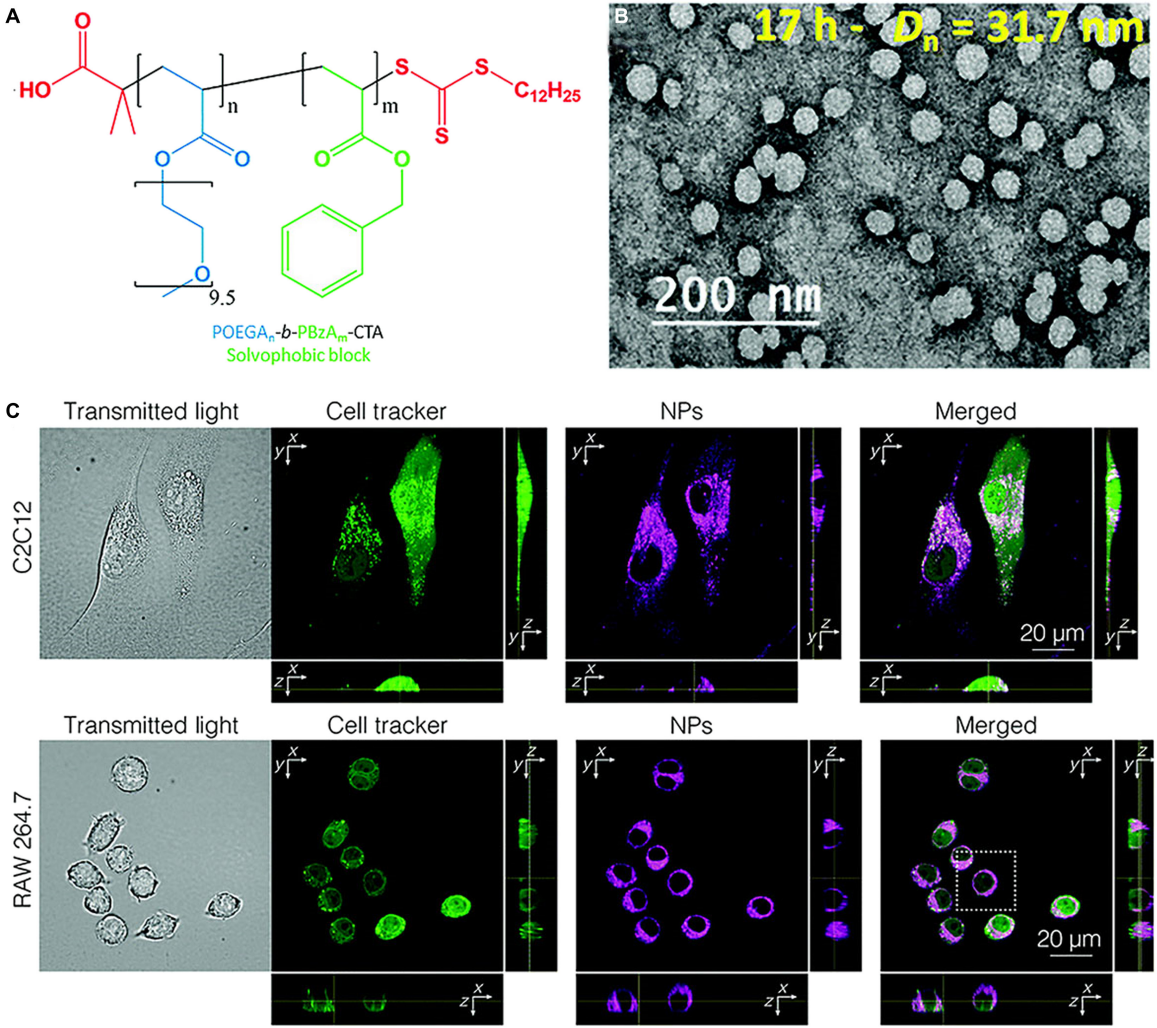
(A) Structure of the amphiphilic NPs prepared by photo-mediated RAFT-PISA. (B) TEM image of POEGA_27_*-b-*PBzA_172_. (C) Cellular uptake of NPs in C2C12 cells and RAW 264.7 cells. Reproduced with permission from [143]. Copyright 2022, Royal Society of Chemistry.

More recently, poly ((oligo(ethylene glycol) acrylate)*-b-*poly(benzyl acrylate) (POEGA*-b-*PBzA) NPs had been fabricated by photo-mediated RAFT-PISA (Fig. 11A) [143]. Notably, sub-50-nm spherical NPs were successfully obtained by the adjustment of chain length (Fig. 11B). Fluorescent molecules were loaded into the NPs through facile swelling procedure. Attractively, the fluorescent sub-50-nm NPs had quantum yield higher than 50% in various solvents. Besides, characterized by the cytotoxicity investigations and the preliminary cell uptake assay, these NPs possessed high biocompatibility and great potential for bioimaging. Confocal fluorescence microscopy images showed that various cells had fluorescent signals after being incubated with the NPs, and these imaging results by NPs and cell tracker were consistent with each other, which proved the feasibility of the fluorescent NPs in biological imaging (Fig. 11C). Notably, although the concentration of dye was 15 nM in water, the higher intracellular fluorescence could be detected. Moreover, the process of polymeric NPs penetrating across cell membrane was observed, and the fluorescence intensity could be monitored within 22 s after injection. In addition, cytoplasmic vesicles (e.g., endosomes and lysosomes) had a higher fluorescence intensity compared with cytosol and nucleus.

**Fig. 12.**
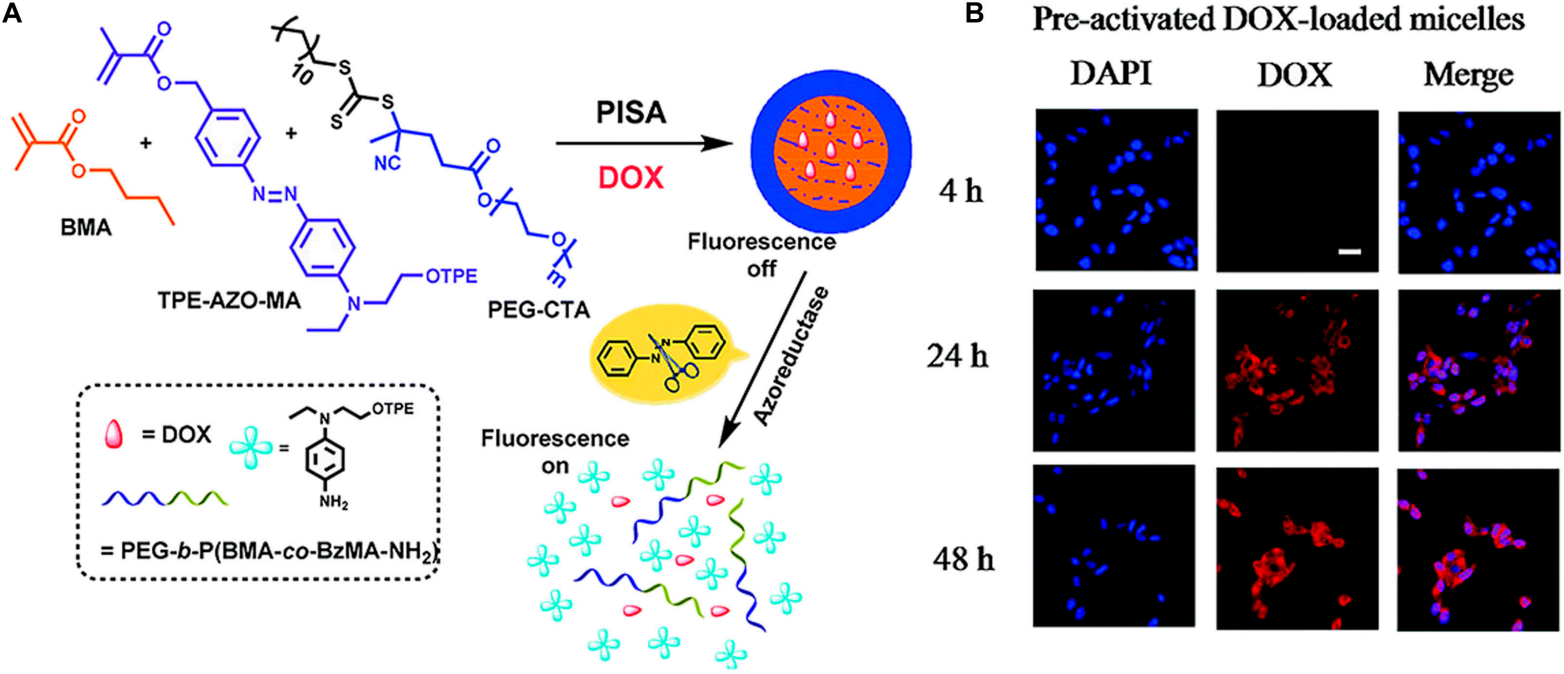
(A) Schematic illustration of the fabrication of smart NPs consisting of PEG*-b-*P(BMA*-co-*TPE-AZO-MA) and the process of DOX release and fluorescence changes. (B) CLSM images of CT26 cells treated with preactivated DOX-loaded micelle solution (40 mg ml^−1^) for 4, 24, and 48 h. Reproduced with permission from [144]. Copyright 2020, Royal Society of Chemistry.

What is more, azo-reductases are commonly found in the human colon, which drives azobenzene-based polymeric NPs to apply in colon disease treatment. A series of tetraphenylethylene (TPE)-azobenzene fluorescent probe-incorporated NPs had been fabricated by RAFT-PISA, while doxorubicin (DOX) was encapsulated in the self-assembly process. Then, the obtained NPs were used for bioimaging and treatment (Fig. 12A) [144]. When these NPs were exposed to azo reductase, the azo double bond would be destructed, leading to the disassembly of NPs and further release of DOX. Notably, the cumulative release of DOX reached 58.5% at 24 h. Instead, only a very small percentage (<10%) of DOX was released without the presence of azoreductase. In addition, the cleavage of azo bond eliminated the fluorescence resonance energy transfer (FRET) process. Therefore, a gradual increase in luminescence intensity was observed within 24 h. The DOX-loaded micelles were first incubated in the presence of enzyme azoreductase for 24 h and then used in cell uptake experiment. After 4 h of incubation, the DOX fluorescent signal was gradually enhanced, indicating that DOX successfully entered cell nuclei and achieved accumulation (Fig. 12B). The obvious fluorescent signal from TPE fragment was also observed after 4 h of incubation. Notably, the obtained NPs with integrated diagnostic and therapeutic functions showed great potential in biomedical applications. Similarly, a novel near infrared (NIR) fluorescent nanoprobe was designed to realize synchronous drug release and cell imaging [145]. The NIR fluorophore Aza-BODIPY moiety was introduced into the hydrophilic macro-CTA and chain-extended with the hydrophobic monomer BzMA to fabricate spherical and vesicular NPs. In a simulated colon environment, reduction-triggered drug release and real-time fluorescent behavior of these obtained NPs were investigated. With the extension of incubation time up to 24 h, the gradual release behavior of DOX (~60%) was observed. In the process of incubation, the NPs gradually dissociated; thus, the aggregation-caused quench (ACQ) effect disappeared and increased fluorescence intensity was observed. Then, the fluorescent NPs were incubated with mouse colon cancer cells (CT26 cells) under normoxia (16% O_2_) and hypoxia (1% O_2_) to test cell imaging. Compared with the normoxic environment, higher fluorescence intensity was observed in the hypoxic environment, which could explain the overexpression of azoreductases in hypoxic cancer cells. In general, the NIR fluorescent nanoprobes can be used to build an integrated platform for diagnosis and treatment.

In conclusion, polymeric NPs constructed by the PISA method have been used in bioimaging. However, owing to the strict requirements for solvents and monomers, current NPs are far from satisfaction. Furthermore, more suitable PISA formulations to improve the biocompatibility of NPs are also urgent. In fact, the significant values of encapsulation of imaging agents for bioimaging are worthy of more research work.

### Disease treatment

**Fig. 13.**
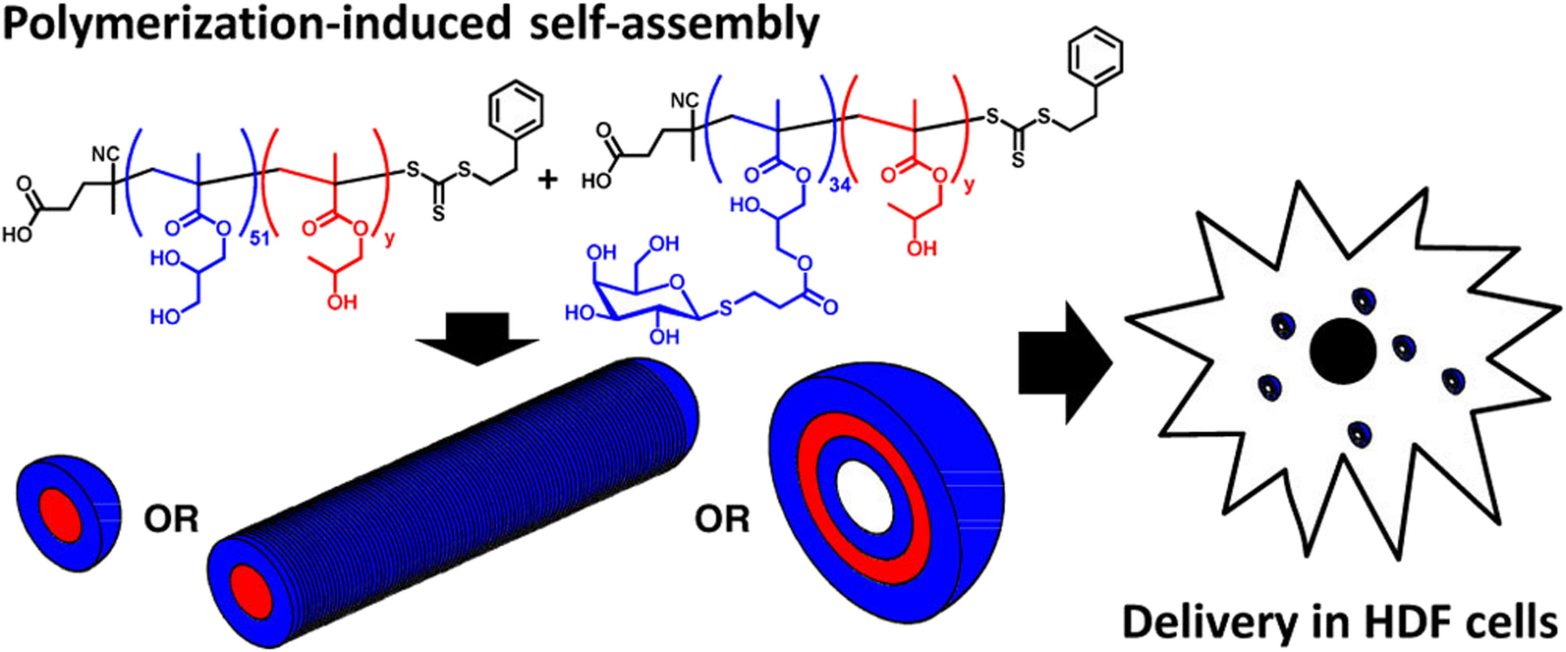
Drug delivery system constructed by PISA. Reproduced with permission from [146]. Copyright 2013, American Chemical Society.

As commonly used cancer treatment, chemotherapy, where small molecule drugs kill cancer cells, usually causes serious side effects to patients. Meanwhile, the nonspecific distribution of therapeutic drugs is another obstacle. Nowadays, nanocarriers have been widely used to improve the solubility and pharmacokinetics of therapeutic drugs. Generally, extended blood circulation time and targeted accumulation of these drugs can be achieved by encapsulation in polymeric NPs. As mentioned, PISA is emerging as a robust technique to fabricate suitable nanocarriers for encapsulation of therapeutic drugs. Plenty of NPs have been exploited for intracellular delivery [146–149], proving that PISA has great potential in constructing drug delivery systems (Fig. 13).

#### Small drug delivery system

For example, poly[oligo(ethyleneglycol) methacrylate]-*b*-[poly(styrene)*-co-*poly(vinyl benzaldehyde)] [POEGMA*-b-*P(ST*-co-*VBA)] NPs with various morphologies were prepared by RAFT-PISA [150]. DOX was successfully encapsulated in the core of NPs through conjugation with aldehyde groups via pH-breakable bonds, and the drug loading efficiency reached 67%. Most drugs (~80%) could be released from nanocarriers at pH 5.0 in a controlled manner, which was much higher than that at pH 7.4. Subsequently, the cellular uptake behaviors of these DOX-loaded NPs were investigated by incubating with breast cancer cells (MCF-7) for 24 h. Notably, during the incubation process, the highest cell uptake efficiency was observed for worm- and rod-like structures by using flow cytometry. The final result could explain that the higher aspect ratio of worm- and rod-like structures caused a greater adhesion of polymeric NPs to the cell membrane compared with vesicle and spherical micelles. In addition, the half maximal inhibitory concentration (IC_50_) of the worm- and rod-like structures was 7 times lower than spherical micelles. The above result was consistent with the higher cell uptake of the worm- and rod-like NPs. Similarly, Pan and coworkers also explored the influence of morphology on drug delivery processes [151]. Briefly, poly((*N,N*-dimethylamino)ethyl methacrylate) (PDMAEMA) was used as macro-CTA to polymerize with *p*-(methacryloxyethoxy)benzaldehyde (MAEBA) units for in situ fabrication of polymeric NPs. The aldehyde groups of PMAEBA were used to conjugate with DOX. Afterward, HeLa cells were incubated with these NPs with various morphologies (e.g., spheres, nanorods, and vesicles) for 24 h. The results by testing the fluorescent intensity of DOX indicated that the cellular uptake rate had the following order: vesicle > nanorod > sphere. However, the in vitro cytotoxicity of DOX-loaded nanorod was more cytotoxic than the DOX-loaded vesicle, which demonstrated that more DOX molecules were released from the DOX-loaded nanorods. Relatively more nanorods than vesicles were localized in acidic organelles, and the drugs were more likely to be released in acidic conditions. These above results may guide the future design and fabrication of drug delivery nano-system and improve the effectiveness of the disease treatment.

As known, drug loading efficiency is a key parameter for the encapsulation of drugs. Recently, the introduction of coordination interactions between polymers and chemo drugs is an efficient strategy to achieve high drug loading efficiency [152]. In addition, design and fabrication of prodrug monomers is also an ideal strategy to improve drug loading efficiency [153–155]. During PISA, therapeutic prodrugs can be directly conjugated to polymeric backbones. For example, the camptothecin (CPT)-containing copolymer PEG*-b-*P(2-(2-methoxyethoxy)ethyl methacrylate PEG-*b*-P(MEO_2_MA)*-co-*CPTM) was used as macro RAFT agent to polymerize with BzMA monomers to achieve prodrug NPs [154]. It was worth mentioning that this method realized polymerization, self-assembly, and drug loading simultaneously. Therefore, the obtained prodrug NPs did not need complex purification, saving a lot of time and labor. Notably, the drug content could be facilely tuned by adjusting the molar ratio of CPTM/MEO_2_MA. Notably, encapsulated CPT could be released under reductive environment. The obtained prodrug NPs were conducted with in vitro drug release at different concentrations of glutathione (GSH) (0.01, 5, and 10 mM). The higher cumulative CPT release was achieved with the higher GSH concentration. The cumulative CPT release reached 42% after incubation in 10 mM GSH for 48 h. Notably, the obtained polymeric NPs without CPT conjugation were noncytotoxic even when the concentration was up to 1,000 μg/ml. To further study the intracellular drug release of the prodrug micelles, CLSM results demonstrated that the CPT drug was gradually released and accumulated in cell nucleus. In general, PISA provides a robust method to construct CPT prodrug NPs, facilitating the development of the field of nanodrug delivery.

Recently, to overcome the disadvantages of small conventional platinum analogs and the limitations of traditional assembly methods, one approach was used to use ROMP-PISA in water to incorporate platinum-based drug into polymeric NPs [156]. In brief, the poly(oligo ethylene glycol) (POEG) stabilizer was directly chain-extended with the cisplatin analog norbornene dicarboximide (CAND) for the fabrication of drug-loaded NPs. Meanwhile, pH-responsive 2-(diisopropylamino)ethyl norbornene dicarboximide (DEND) monomers were used to achieve controllable disassembly upon acid tumor environment. The effect of the size and zeta potential on cell uptake procedure was systematically studied. Among these platinum-loaded NPs, smaller NPs had higher cytotoxicity, while positively charged NPs had higher cell uptake efficiency than the negatively charged ones, facilitating the design of NPs with better performance. In addition, owing to the ability of preparing platinum-loaded NPs in high concentration, ROMP-PISA promises to formulate pharmaceuticals for low volume injections at high dose for human disease.

#### Macromolecular drug delivery system

Besides small therapeutic drugs, proteins are specific and highly active substances, which play an important role in regulation of vital activities. In recent years, much effort has been devoted to deliver therapeutic proteins for precise nanomedicine. However, owing to its poor stability and short circulation time in vivo, the application prospects are greatly limited.

**Fig. 14.**
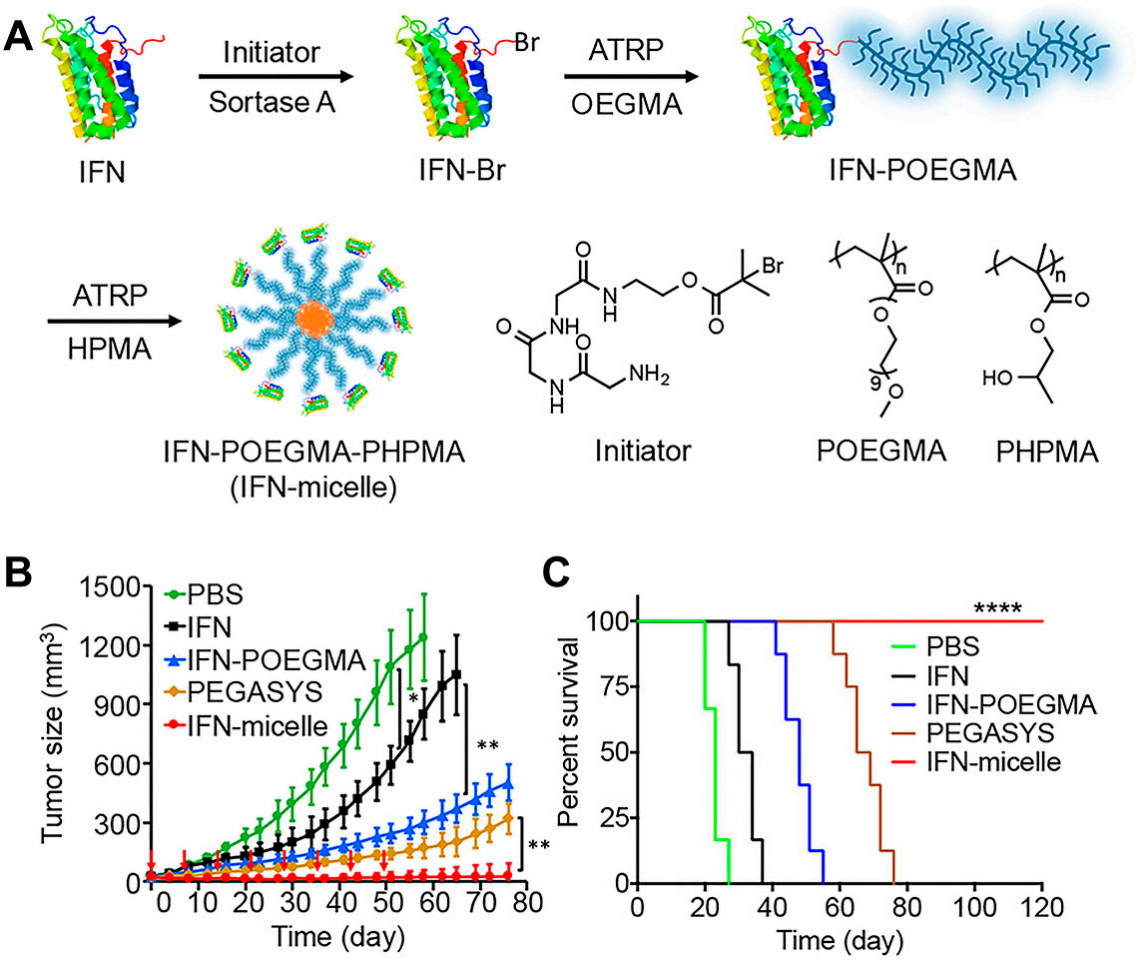
(A) Schematic diagram for the illustration of the IFN-POEGMA-PHPMA micelle fabricated by ATRP-PISA. (B) Tumor growth curves after treatment with different formations. (C) Cumulative survival of mice with different treatments. Reproduced with permission from [159]. Copyright 2018, American Chemical Society.

The use of therapeutic proteins in the clinic has been increasing quickly in the past few years because of high efficiency [157,158]. Unfortunately, the stable delivery of proteins is quite difficult because proteins are susceptible to immune responses and are easily inactivated upon internal or external conditions. Normally, modification of proteins by using hydrophilic chain segments can extend their in vivo blood circulation time. For example, the PEGylated interferon-α (PEGASYS) has 8.6-fold half-life time than native therapeutic protein interferon-α (IFN). What is more, Gao and coworkers improved the in vivo half-life time of IFN as long as 83.8 h by site-specific in situ PISA (SI-PISA) strategy (Fig. 14A) [159]. However, PEGylation products usually have lower bioactivities owing to the presence of positional isomers. Here, to avoid creating positional isomers, POEGMA was used as an alternative to PEG. Compared with IFN, the plasma IFN levels of IFN-micelle consisted of IFN-POEGMA-PHPMA decreased slowly after intravenous injection. Then, the biodistribution of IFN-micelle was studied in the ovarian tumor-bearing mouse model. The IFN levels of IFN-micelle had significantly increased accumulation in major organs compared with PEGylation and POEGMA conjugation of IFN. Furthermore, the IFN-micelle was more effective than PEGASYS in terms of inhibiting tumor growth (Fig. 14B). More significantly, after treatment with IFN-micelle, the survival rate of mice remained 100% as long as 120 days (Fig. 14C). The above results indicated that the SI-PISA has great potential to improve the pharmacology of therapeutic proteins.

However, the chemical modifications of proteins may cause some changes to their inherent characteristics. Therefore, physical encapsulation of the protein is also a good choice. Traditional method of encapsulating therapeutic protein usually requires multiple steps, and the polymeric vesicles need to be permeable. l-Asparaginase (ASNS) is a therapeutic enzyme used for leukemia treatment, which has been encapsulated in vesical cavity by the one-pot method [160]. The successful preparation of ASNS-loaded vesicles by aqueous photo-PISA has been characterized by TEM, atomic force microscope (AFM), and DLS methods. The mild reaction condition of photo-PISA did not disrupt the structure of proteins. A greater stability in vitro and in vivo had been achieved compared to native protein or PEGylated conjugate. Besides, the antibodies would be blocked from entering the vesicle. Thus, the immunogenicity of the enzyme was reduced under the protection of vesicles, lowering the risk of an immune response. Notably, the in vitro experiments showed that cell proliferation suffered from enhanced inhibition when treated with ASNS-loaded vesicles (58%) compared to PEG-ASNS (61%) and free ASNS (78%). Finally, ASNS encapsulated in the polymeric vesicles has lower accumulation in the liver and the kidneys compared to free ASNS. Therefore, the size-selectively permeable vesicle prepared by PISA is a good choice to encapsulate protein without chemically altering the protein, thus providing better therapeutic effect.

More recently, an innovated dengue virus-mimicking triblock vesicle was designed to treat triple-negative (TN) breast cancer [161]. Here, poly(glycerol monomethacrylate) (PGMA) as macro-CTA was successively chain-extended with HPMA and 2-(diisopropylamino)ethyl methacrylate (DPA) monomers. The protonation behavior of PDPA block under acid environment endowed the vesicles with ultra pH-responsive behavior. To achieve active targeting property, a small fraction (3 mol%) of the PGMA was replaced by poly(2-(methacryloyloxy)ethyl phosphorylcholine) (PMPC), which could actively target SR-B1 scavenger receptor-overexpressed TN breast cancer cells. In cell uptake experiments, when treated with vesicles without PMPC block, all cells showed low uptake. However, triblock copolymer vesicles containing PMPC block could actively target TN breast cancer cells. The obtained vesicles were used to load plasmid DNA (pEGFP) by electroporation and then used in the cell uptake experiments to prove the ability to target the nuclear region. By characterizing the expression of fluorescent EGFP within the cells, the vesicle could deliver functional biomolecules (e.g., plasmid DNA) to the nuclear region. Finally, the bionic vesicles constructed by PISA can provide better therapeutics for TN breast cancer.

What is more, heparin is a sulfated polysaccharide, having great potential in wound healing and tissue regeneration by stabling the fibroblast growth factors (FGFs). Notably, PISA can also be used to fabricate heparin-mimicking sulfonated NPs. For this case, poly(2-acrylamido-2-methyl propane sulfonic acid) [P(AMPS)] was used as macro-CTA to chain-extend with St units [162]. The NPs showed low biotoxicity to murine embryonic fibroblast cells even if the concentration reached 1 mg ml^−1^. Also, the heparin-mimicking NPs displayed greater cellular proliferation than heparin (400% versus 150%). The difference of cellular proliferation may be mainly attributed to the existence of numerous surface-active P(AMPS) chains at the surface of NPs, thus promoting the stabilization of FGF. In general, PISA provides a facile route to fabricate sulfonated NPs at high solid content, thus facilitating the study of heparin mimicry.

### Biocatalysis

**Fig. 15.**
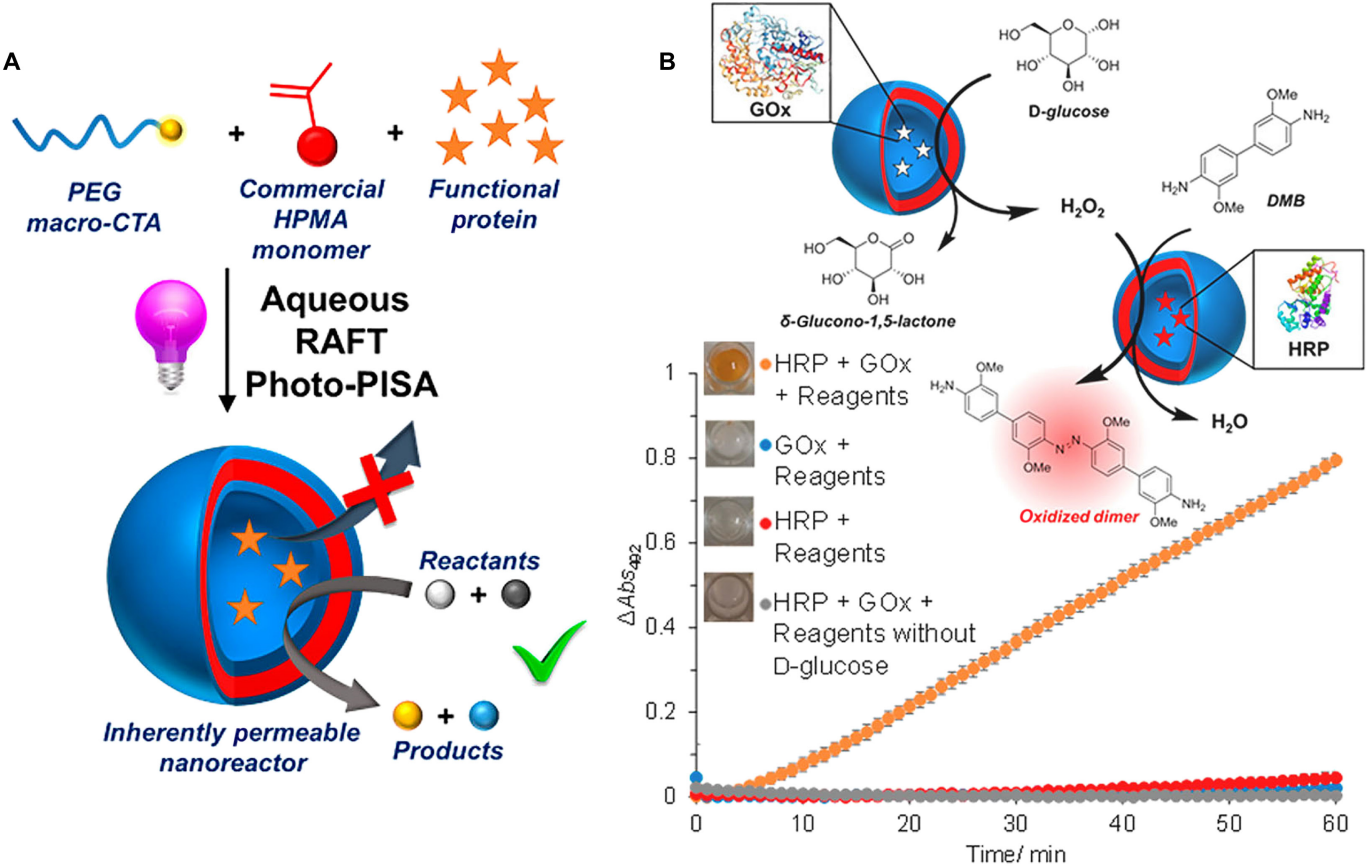
(A) Schematic illustration of permeable protein-loaded nanoreactor prepared by aqueous RAFT-PISA. (B) Illustration for the study on the activity of cascade reaction between HRP and GOx-loaded vesicles. Reproduced with permission from [164]. Copyright 2017, American Chemical Society.

Enzymes have received significant attention for the high efficiency in biocatalysis [163]. However, enzymes are prone to be inactivated as a result of being stimulated by external environment. The nanoreactor can isolate the protein from the external environment and enable the protein to interact with small substrates simultaneously. In 2017, O’Reilly and coworkers reported the polymeric vesicles consisting of PEG*-b-*PHPMA prepared by aqueous RAFT photo-PISA (Fig. 15A) [164]. In the paper, the permeable nature of the PHPMA membrane was discussed for the first time. The PHPMA membranes were highly hydrated; thus, small molecules could get in and out of vesicles, while the enzymes were confined in the cavities. A range of functional enzymes, such as HRP and GOx, had been successfully encapsulated into polymeric vesicles, and the enzymes also remained active owing to the permeability of the membrane. GOx consumed d-glucose, simultaneously producing δ-glucono-1,5-lactone and H_2_O_2_ as accessory substance. Then, the produced H_2_O_2_ was used to catalyze the oxidation of 3,3′dimethoxybenzidine (DMB) to a colored dimer product by HRP. Only when both enzymes and reagents existed, cascade reaction can be observed (Fig. 15B). Furthermore, cascade reaction between HRP and GOx proved the permeability of the PHPMA membrane.

Later, the same group adopted the cross-linked strategy to adjust the membrane permeability of nanoreactors. First, well-defined enzyme-loaded vesicle that self-assembled by PEG*-b-*P(HPMA*-co-*GlyMA) BCPs was prepared at ambient reaction condition by photo-PISA [165]. The pendant epoxide groups of PGlyMA units in the hydrophobic layer provided the opportunity to react with amino groups to achieve cross-linking effect. Next, various kinds of primary amines, such as PEG diamines and linear aliphatic diamine, were used to cross the membranes of these vesicles. However, the obtained enzyme-loaded vesicles all showed decreased enzyme activity, which was ascribed to decreased membrane permeability. Among them, the vesicle cross-linked by hydrophobic amines could reduce permeability up to 80%. In general, the increased thickness and reduced permeability as a result of membrane cross-linking adjusted the enzyme activity by controlling the diffusion process of substrates. This study provided a robust strategy to tune the membrane permeability, which may have irreplaceable guiding significance on the future design of nanoreactors.

Although changing the DP of hydrophobic block or copolymerizing with other monomers can adjust the membrane permeability, the vesicle morphology may also change. Recently, well-defined copolymer vesicles with tunable membrane thicknesses and compositions prepared by aqueous seeded photo-PISA were used as enzymatic nanoreactors [166]. By copolymerizing with HPMA or other functional monomers, vesicles with various compositions and membrane thicknesses could be successfully fabricated, without changing the vesicle morphology. Notably, given the size-selective permeability of the PHPMA membrane, the HRP-loaded vesicles could oxidize 2,2′-azinobis (3ethylbenzothiazoline-6-sulfonic acid) (ABTS) into ABTS^+^ in the presence of H_2_O_2_. By tuning membrane thickness or hydrophobicity, the enzymatic reaction rate could be finely adjusted.

### Antimicrobial

**Fig. 16.**
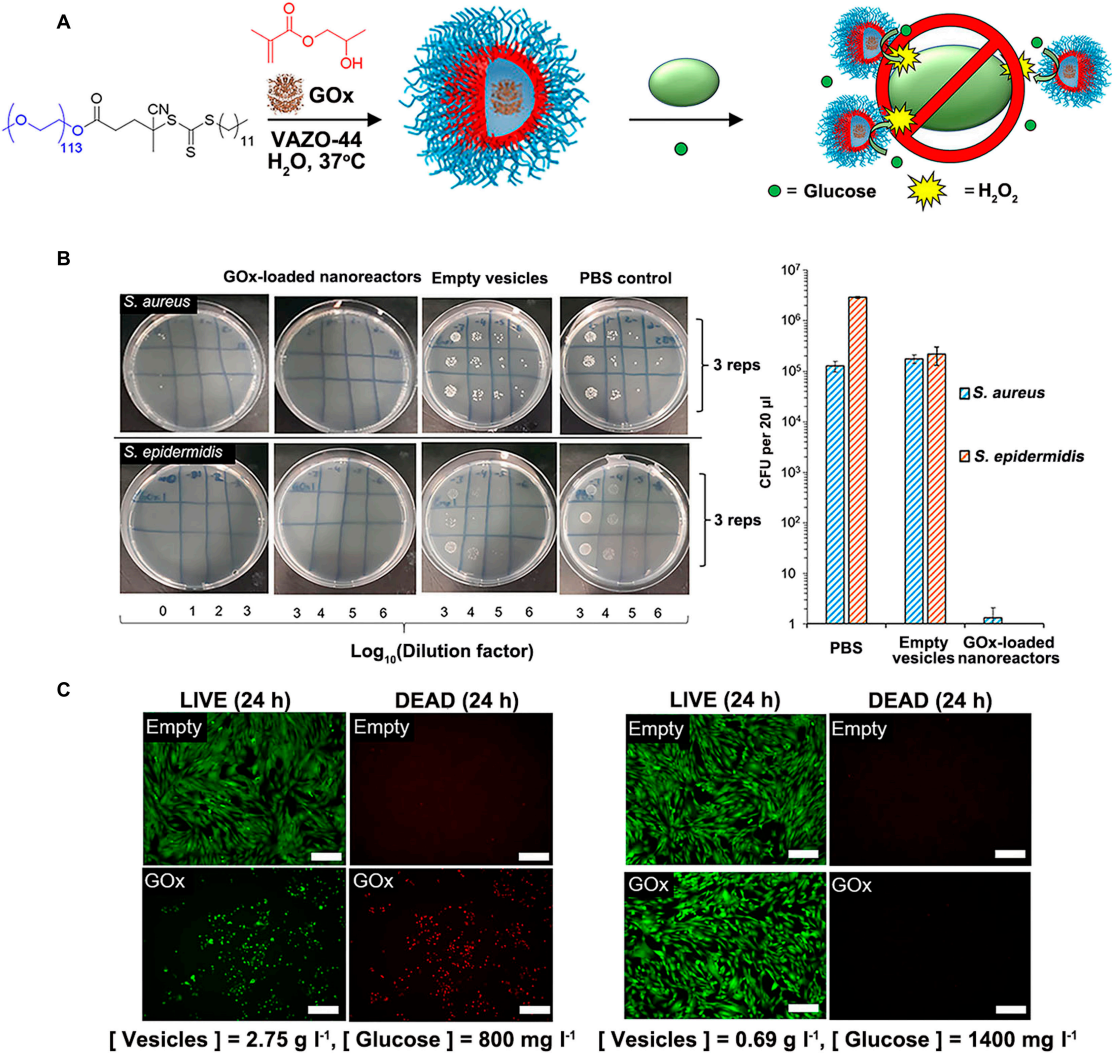
(A) Schematic illustration of the GOx-loaded PEG_113_-PHPMA_400_ nanoreactors prepared by aqueous PISA and the antimicrobial action in the presence of glucose. (B) The activity comparison of *S. aureus* and S. *epidermidis* after 24-h incubation of GOx-loaded nanoreactors and empty vesicles. (C) The biotoxicity changes of GOx-loaded nanoreactors and empty vesicles at 2.75 mg l^−1^ or 0.69 mg l^−1^. Reproduced with permission from [169]. Copyright 2020, American Chemical Society.

In recent years, the number of novel antibiotics entering the clinic is gradually decreasing, and the threat of antibiotic resistance is gradually increasing. Based on the forecast, the bacterial infections will become the leading cause of death in the near future [167,168]. Therefore, novel and effective antimicrobial agents have been explored to fight against bacterial infections. Therein, combining antimicrobial enzymes and polymeric nanoreactors is a potential method to overcome microbial infections. In the past, honey was used as natural antimicrobial medicine, which is contributed to the H_2_O_2_ generated by GOx in honey. Encapsulation of GOx into polymeric nanoreactors can maintain the activity of enzyme, thus providing antibacterial properties. The PEG_113_-*b*-PHPMA_400_ vesicles were prepared by thermal-initiated aqueous RAFT-PISA at 37 °C, and GOx was encapsulated during the assembly process with an encapsulation efficiency of about 24% (Fig. 16A) [169]. The catalytic activity of nanoreactors was characterized under various glucose concentrations, in which higher glucose concentration produced more H_2_O_2_. *Staphylococcus aureus* and *Staphylococcus epidermidis* were chosen to investigate the antimicrobial properties of GOx-loaded nanoreactors. When incubated with the GOx-loaded nanoreactors for 24 h, the growth of 2 Gram-positive staphylococcal strains was inhibited (Fig. 16B). According to colony-counting assay, the GOx-loaded nanoreactors reduced bacterial growth by 5 logs for S. *aureus* and 6 logs for S. *epidermidis* in the presence of 170 mg l^−1^ glucose concentration. Furthermore, the antibacterial universality of GOx-loaded nanoreactors was also investigated by conducting antibacterial tests with *Escherichia coli* and *Klebsiella pneumoniae*. Notably, the growth of *K. pneumoniae* could be reduced at 800 mg l^−1^ glucose concentration. However, the reduced growth of *E. coli* was achieved at 5,170 mg l^−1^ glucose concentration, which was far higher than the normal glucose concentration in human body. In general, the antimicrobial property of GOx-loaded nanoreactors was highly effective for most strains of bacteria. In addition, in view of the sensitivity of fibroblasts to H_2_O_2_, the concentration of GOx-loaded nanoreactors could be decreased to 0.69 mg ml^−1^ (Fig. 16C).

As known, short cationic peptides can combine with phospholipid head-groups on the bacterial membranes and further promote cell death. Therefore, most antimicrobial peptides (AMPs) are cationic peptides. Similar to small molecule drugs, AMPs also suffer from poor stability, salt sensitivity, and toxicity. Therefore, AMP-containing NPs can be prepared by PISA to overcome the above limitations. The polylysine macro-CTA was chain-extended with HPMA monomers under aqueous condition to prepare NPs with various morphologies [170]. Furthermore, the various NPs were used to investigate the antimicrobial property of Gram-positive bacterium. The experimental results showed that the spherical NPs consisting of polylysine-*b*-PHPMA_12_ had a 99.96% bacteria removal, which was significantly high compared with worms and vesicles. The higher AMP content and the larger specific surface area of spherical NPs were the main reasons for the result. Notably, the polylysine-*b*-PHPMA_12_ NPs could be used to prepare film membranes, and then the membranes were applied to filter the water containing *S. epidermidis*.

## Conclusion and Future Perspective

Over the past decade, with the development of polymerization technology, a range of monomers can be applied to PISA for the preparation of NPs. Notably, PISA has shown many advantages in fabrication of various nanoplatforms especially in terms of high efficiency and labor-saving. Among the various PISA formulations, RAFT-PISA is mostly investigated and used. The related reaction mechanism and morphology control have been thoroughly studied. However, nondegradable carbon–carbon skeletons and the biotoxicity of thioester functional groups are not good for biomedical application. Other PISA formulations (e.g., NMP-PISA and ATRP-PISA) can provide complement to RAFT-PISA. Especially, NCA-PISA is an emerging PISA approach. Considering facile synthesis and excellent biocompatibility of polypeptides, some pioneering work has already been reported. The biomedical nanoplatforms fabricated by PISA have been used in the field of bioimaging, disease treatment, biocatalysis, and antimicrobial. As a robust technique to scale up preparation of NPs, PISA is able to encapsulate functional molecules for various applications. However, the development of functional nanoplatforms by PISA is still in its infancy, and many issues should be solved in the future.

1. The types of monomers suitable for PISA are worthy of more exploration. In recent years, with the development of controllable polymerization technology, more monomers can be used in PISA formulations. However, compared with traditional self-assembly method, PISA formulations should consider the solubility of the monomer and the choice of solvent, which resulted in a relatively small number of suitable monomers. More specifically, a range of functional BCPs have been designed to construct nanoplatforms by traditional self-assembly. In comparison, the nanoplatform constructed by PISA still needs further improvements. Therefore, it is necessary to expand the scope of monomers. In consideration of the high efficiency of PISA method, more efforts should be used.

2. Current biomedical nanoplatforms constructed by PISA are relatively simple and very limited. More biology-related experiments should be conducted to prove the great potential of PISA in fabricating biomedical platforms. In additional, most of the studies are based on RAFT-PISA, which may bring biotoxicity into polymeric nanoplatforms. There are convenient ways to eliminate the end groups of CTAs, which undoubtedly introduce complexity into the PISA process. Notably, emerging NCA-PISA that has both high biocompatibility and mild preparation is extraordinary. Therefore, exploring more NCA monomers that have stimuli-responsive characteristics can be taken into account and may boost future development of PISA.

3. The encapsulation efficiency of functional molecules by PISA is still challenging. For biomedical applications, the current approach is to physically encapsulate these molecules in the cavities of fabricated vesicles. However, the encapsulation efficiency is unsatisfactory and the purification is complex. In addition, the release of encapsulated molecules is also not controllable in most PISA instances. In fact, pH response, enzyme response, light response, magnetic response, and ultrasonic response can be introduced into the PISA system to control local release. Therefore, designing prodrug monomers or conjugating functional molecules to monomers via stimuli-responsive linkages are worth considering. This strategy not only improves the encapsulation efficiency but also allows for controlled release.

4. The broad application of PISA needs to be further explored. In addition to its biomedical application, PISA can also be applied in the field of nanocomposites, functional coatings, hydrogel, and so on. PISA technology has a significant potential in a variety of application areas. Although the PISA technology has been developed for many years, its application is still in the exploration and preliminary stage. Therefore, more effort should be put to enrich the application of PISA.
